# Osteoblasts secrete Cxcl9 to regulate angiogenesis in bone

**DOI:** 10.1038/ncomms13885

**Published:** 2016-12-14

**Authors:** Bin Huang, Wenhao Wang, Qingchu Li, Zhenyu Wang, Bo Yan, Zhongmin Zhang, Liang Wang, Minjun Huang, Chunhong Jia, Jiansen Lu, Sichi Liu, Hongdong Chen, Mangmang Li, Daozhang Cai, Yu Jiang, Dadi Jin, Xiaochun Bai

**Affiliations:** 1 Academy of Orthopedics, Guangdong Province, Department of Orthopedics, The Third Affiliated Hospital, Southern Medical University, Guangzhou 510630, China; 2 State Key Laboratory of Organ Failure Research, Department of Cell Biology, School of Basic Medical Science, Southern Medical University, Guangzhou 510515, China; 3 Department of Pharmacology and Chemical Biology, University of Pittsburgh School of Medicine, Pittsburgh, Pennsylvania 15213, USA

## Abstract

Communication between osteoblasts and endothelial cells (ECs) is essential for bone turnover, but the molecular mechanisms of such communication are not well defined. Here we identify Cxcl9 as an angiostatic factor secreted by osteoblasts in the bone marrow microenvironment. We show that Cxcl9 produced by osteoblasts interacts with vascular endothelial growth factor and prevents its binding to ECs and osteoblasts, thus abrogating angiogenesis and osteogenesis both in mouse bone and *in vitro*. The mechanistic target of rapamycin complex 1 activates Cxcl9 expression by transcriptional upregulation of STAT1 and increases binding of STAT1 to the *Cxcl9* promoter in osteoblasts. These findings reveal the essential role of osteoblast-produced Cxcl9 in angiogenesis and osteogenesis in bone, and Cxcl9 can be targeted to elevate bone angiogenesis and prevent bone loss-related diseases.

## Abstract (web summary)

Bone development and vascularization are coupled events that share many molecular mechanisms. Here the authors identify osteoblast-secreted Cxcl9 as an inhibitory regulator of angiogenesis and osteogenesis, and show that mTORC1 signaling and STAT1 are critical upstream mediators of the cytokine expression.

During development of the mammalian skeleton, the formation of endochondral bone coincides with capillary invasion^1^,^2^, suggesting a close connection between osteogenesis and angiogenesis. Blood vessels supply osteoblast precursors either by their flow^3^ or components in their walls^4^,^5^, and guide the migration of osteoblast precursors from the periosteum to the bone marrow^4^. Impairment of angiogenesis decreased trabecular bone formation as well as expansion of the hypertrophic zone into the growth plate^6^. Postnatal bone remodelling also depends on vascular formation. Oxygen, nutrients and a cornucopia of hormones and growth factors, which are important for bone and bone marrow development and homeostasis, are transported to bone by blood vessels^7^,^8^. Recent studies have suggested a direct role of decreased angiogenesis in senile and postmenopausal osteoporosis^9^, highlighting the importance of the regulation of angiogenesis in bone.

Bone marrow is a highly heterogeneous and vascularized tissue. The diverse cell types populating the bone marrow communicate extensively with each other, and the cell-to-cell cross-talk is vital for correct bone development and homeostasis^10^. The cross-talk between bone-forming osteoblasts and vessel-forming endothelial cells (ECs) is progressively gaining strong support in the scientific community^11^. In particular, osteoblasts secrete angiogenic factors, such as vascular endothelial growth factor (VEGF)^12^ and erythropoietin^13^, to mediate the cross-talk between osteoblasts and ECs. However, molecules that couple osteoblasts and ECs to modulate bone remodelling and angiogenesis have not been fully defined, and the signalling pathways that control the production of these molecules in osteoblasts are unclear.

The mechanistic target of rapamycin complex 1 (mTORC1) integrates diverse intracellular and extracellular signals^14^ and plays a central role in the regulation of cell growth, proliferation and metabolism^15^. Activation of mTORC1 enhances VEGF synthesis to promote angiogenesis in tumours^16^. Although recent studies have defined mTORC1 signalling as a critical regulator of osteoblastogenesis and bone formation^17^,^18^, the role of mTORC1 in bone vessel formation is unknown.

In this study, we found that mice with constitutive mTORC1 activation in osteoblasts demonstrated enhanced VEGF secretion, but unexpectedly decreased phosphorylation of its receptor (VEGFR2) and downstream signalling in ECs, and markedly reduced vasculature formation in bone. We further identified a CXC-chemokine, chemokine (C-X-C motif) ligand 9 (Cxcl9) as a direct counter-regulatory molecule of VEGF signalling constitutively produced by osteoblasts to suppress angiogenesis and osteogenesis in bone. Mechanistically, the mTORC1 activated Cxcl9 expression by transcriptional upregulation of STAT1 and increased binding of STAT1 to the *Cxcl9* promoter in osteoblasts. Thus, our study identified Cxcl9 as an angiostatic factor secreted by osteoblasts, supporting Cxcl9 as a novel target for stimulating angiogenesis and osteogenesis in bone.

## Results

### Osteoblastic mTORC1 regulates bone angiogenesis

**Figure 1:**
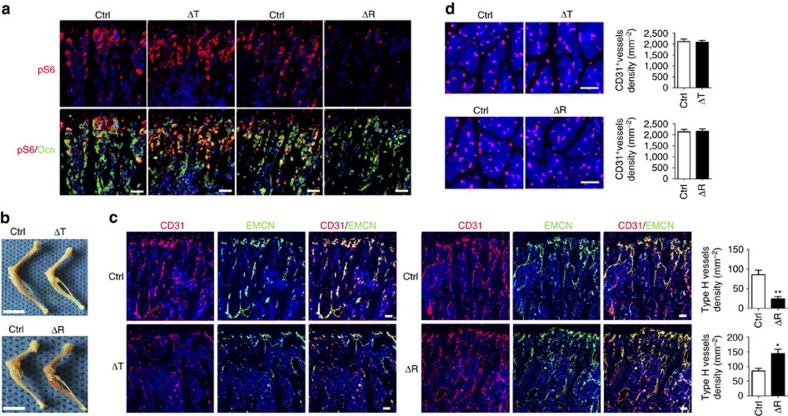
Alteration of mTORC1 activity in osteoblasts affects angiogenesis in mouse bone. (**a**) Representative images of immunostaining of pS6 (Ser235/236) and osteocalcin (Ocn) in 12-week-old male mice bone. Scale bar, 50 μm. (**b**) Photograph of hindlimbs of 6-week-old male Δ*Tsc1* (ΔT) and Δ*Raptor* (ΔR) mice and their littermate controls (Ctrl). Scale bar, 1 cm. (**c**) Representative images of CD31^+^EMCN^+^ microvessels and quantitative analysis of type H microvessel density in femur sections of 12-week-old male mice. Scale bar, 100 μm. *n*=9 per group. (**d**) Consistent numbers of CD31^+^ vessels in surrounding muscle of mouse bone. Scale bar, 100 μm. Data are shown as mean±s.d. *n*=9 per group. Data are shown as mean±s.d. *P<0.05, **P<0.01 (Student's *t*-test). For all panels in this figure, data are representative for three independent experiments.

Riddle *et al*. and our group reported that activation of mTORC1 in osteoblast lineage cells prevented osteoblast maturation and bone formation^17^,^18^. As osteogenesis and angiogenesis are tightly coupled in bone, we determined whether bone angiogenesis was affected in mice with constitutive mTORC1 activation in osteoblasts. *Osx-cre*^19^ has previously been reported to target other cell types besides osteoblast lineage cells^20^. To achieve specific activation of mTORC1 in osteoblasts, we crossed floxed *Tsc1* (mTORC1 negative regulator) mice^21^ with mice expressing the Cre recombinase driven by an osteocalcin (*OC*) promoter (*OC-Cre*)^22^ to produce mice with *Tsc1* deletion in mature osteoblasts (hereafter referred to as Δ*Tsc1* mice). Six-week-old Δ*Tsc1* mice clearly showed enhanced phosphorylation of S6 (Ser235/236) in osteoblasts (positively stained by osteocalcin) (Fig. 1a), indicating that mTORC1 was activated by this genetic manipulation. Micro-computed tomography (micro-CT) analysis revealed the same high volume of immature woven bone in Δ*Tsc1* mice as reported previously^17^, that is, the high bone mass in Δ*Tsc1* mice was the result of increased areas of hypomineralization (Supplementary Fig. 1). At necropsy, we noted pale long bones in Δ*Tsc1* mice (Fig. 1b), indicating reduced blood perfusion in the bone of these mice. We observed a decreased number of CD31^+^Endomucin^+^ vessels, which has been reported to couple angiogenesis and osteogenesis in bone, in tibia sections in Δ*Tsc1* mice when compared with their littermate controls (Fig. 1c). However, number of vessels in surrounding muscle was not affected in Δ*Tsc1* mice (Fig. 1d), suggesting that osteoblasts with hyperactive mTORC1 specifically suppressed vasculature formation in bone.

**Figure 2:**
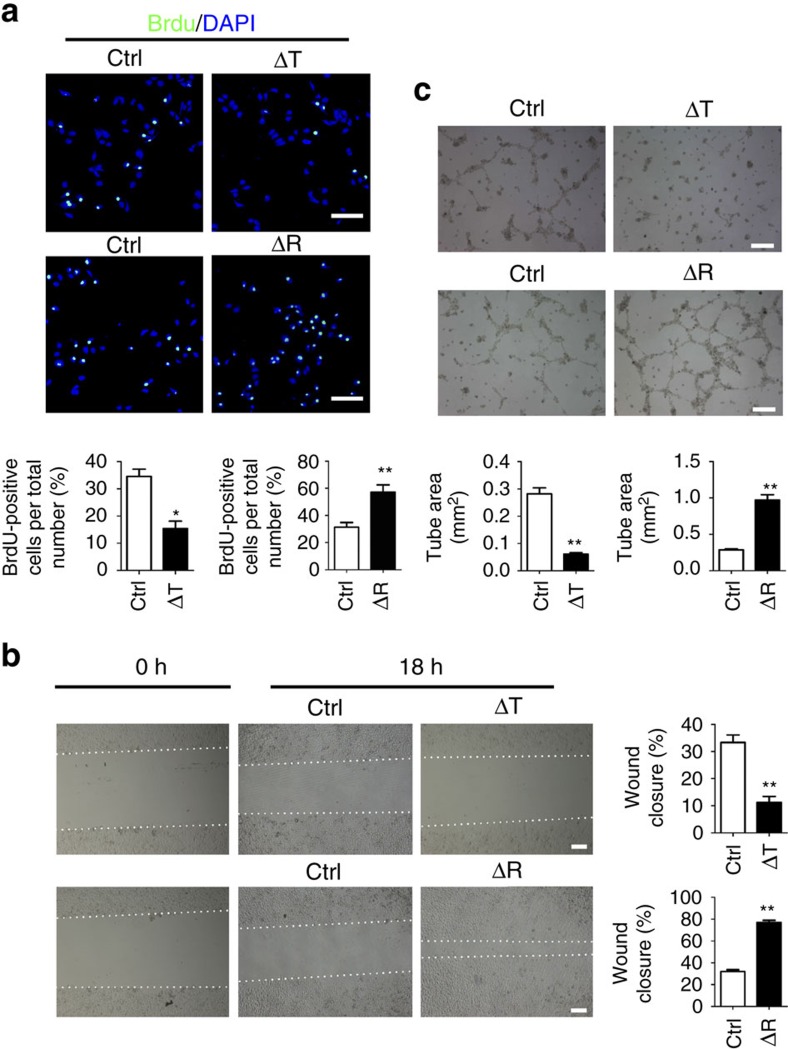
Alteration of mTORC1 activity in osteoblasts affects angiogenesis *in vitro*. (**a**) Representative confocal images of immunostaining of BrdU (green) in HUVECs and quantitative analysis of BrdU^+^ cells over total cells. Scale bar, 50 μm. *n*=9 per group. (**b**) Representative photomicrographs of wounds in HUVECs at 0 h and after 18 h; dotted lines highlight the linear scratch/wound for each group of cells. The bar graph shows the mean percentage of wound closure. Scale bar, 200 μm. *n*=9 per group. (**c**) Representative photomicrographs of tube formation of HUVECs incubated with Matrigel and quantitative analysis of tube area. Scale bar, 200 μm. *n*=9 per group. Data are shown as mean±s.d. *P<0.05, **P<0.01 (Student's *t*-test). For all panels in this figure, data are representative for three independent experiments. Ctrl, control.

To test these observations *in vitro*, we cultured immortalized human umbilical vein ECs (HUVECs) with conditional medium (CM) collected from primary calvarial osteoblasts. HUVECs cultured in CM from Δ*Tsc1* osteoblasts exhibited a lower proliferation and migration rate than those in control CM (Fig. 2a,b). HUVECs seeded on Matrigel in the presence of control medium formed branching, anastomosing tubes, resulting a meshwork of capillary-like structures (Fig. 2c). In contrast, HUVECs remained spherical and isolated when maintained in CM from Δ*Tsc1* osteoblasts, with small cellular nests and short tubes rarely observed (Fig. 2c). These findings suggested that osteoblasts with hyperactive mTORC1 inhibited angiogenesis in mouse bone and *in vitro*.

To definitively establish the role of osteoblastic mTORC1 in regulating angiogenesis in bone, we created mice with mTORC1 inactivation in osteoblasts by crossing the *OC*-Cre mouse with a mouse carrying a floxed *Raptor* (mTORC1-specific component) allele^23^ (control). Immunohistochemical staining of S6 phosphorylation (Ser235/236) confirmed inactivation of mTORC1 in osteoblasts of Δ*Raptor* mice (Fig. 1a). Micro-CT analysis revealed the same lower bone volume in Δ*Raptor* mice as that previously reported^24^ (Supplementary Fig. 2). In contrast to Δ*Tsc1* mice, the long bones from the *Raptor* mutants were more richly perfused with blood compared with control bones (Fig. 1b). Immunohistochemical staining (Fig. 1c) confirmed increased CD31^+^Endomucin^+^ vessel numbers in the bone of Δ*Raptor* mice. However, vessel number in surrounding muscle remained unchanged (Fig. 1d). Consistent with the *in vivo* results, CM from primary Δ*Raptor* osteoblasts induced proliferation (Fig. 2a), migration (Fig. 2b) and network formation (Fig. 2c) of HUVECs *in vitro*. On the basis of these results, we conclude that osteoblasts with impaired mTORC1 promote angiogenesis in bone and *in vitro*.

### Osteoblasts produce angiostatic factors

**Figure 3:**
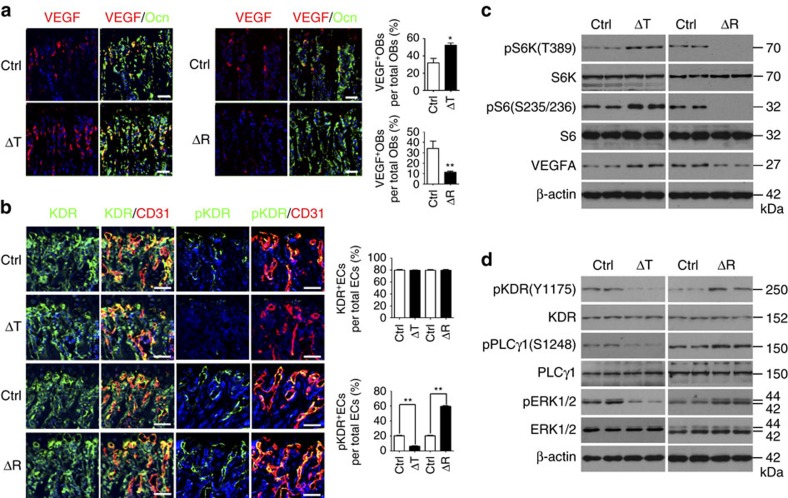
Regulation of VEGF in osteoblasts by mTORC1 does not explain the vascular phenotypes of mutant mouse bone. (**a**) Representative images of immunostaining of VEGF and Ocn in 12-week-old male mice bone and quantitative analysis of VEGF^+^ osteoblasts compared with total osteoblasts. Scale bar, 50 μm. *n*=9 per group. (**b**) Representative images of immunostaining of CD31, KDR and pKDR (Y1175) in 12-week-old male mice bone, and quantitative analysis of KDR^+^ and pKDR^+^ ECs compared with total ECs in bone marrow. Scale bar, 50 μm. (**c**) Western blot of VEGF in primary osteoblasts. (**d**) Western blot of phosphorylation of KDR, PLCγ1 and ERK1/2 in HUVECs treated with CM from primary osteoblasts for 10 min. Data are shown as mean±s.d. *P<0.05, **P<0.01 (Student's *t*-test). Ctrl, control.

The results described above suggested that mTORC1 regulated the expression of angiogenic and (or) angiostatic factors in osteoblasts to modulate the formation of vasculature in bone. Consistent with this notion, enhanced VEGF expression (Fig. 3a) and secretion (Supplementary Fig. 3a) by osteoblasts was observed in Δ*Tsc1* mice, indicating that mTORC1 stimulated VEGF expression in osteoblasts. Nonetheless, the enhanced VEGF expression in osteoblasts, which would be expected to produce more vessels in bone, could not theoretically be responsible for the impaired angiogenesis of bone seen in Δ*Tsc1* mice. VEGFR2 (Kinase insert domain receptor (KDR)), a major receptor transducing VEGF signalling in ECs^25^, exhibited consistent expression, but decreased phosphorylation in ECs when exposed to the overall elevated secretion of VEGF by Δ*Tsc1* osteoblasts (Fig. 3b). In addition, primary Δ*Tsc1* osteoblasts exhibited elevated expression (Fig. 3c) and secretion (Supplementary Fig. 3b) of VEGF *in vitro*, and CM collected from these cells repressed transduction of the VEGF signalling cascade in HUVECs, as revealed by the decreased phosphorylation of KDR and its downstream mediators PLCγ1 and ERK1/2 (Fig. 3d).

The disjoint between VEGF secretion by osteoblasts and VEGF signalling cascade transduction in ECs was copied in Δ*Raptor* mice. Inconsistent with the overall decrease in VEGF expression (Fig. 3a) and secretion (Supplementary Fig. 3a) by Δ*Raptor* osteoblasts, phosphorylation of KDR was enhanced in ECs in the bone marrow of Δ*Raptor* mice (Fig. 3b). Moreover, while Δ*Raptor* osteoblasts expressed (Fig. 3c) and secreted (Supplementary Fig. 3b) reduced VEGF *in vitro*, HUVECs showed enhanced VEGF signal transduction when cultured in CM from Δ*Raptor* osteoblasts (Fig. 3d). Together, these data suggested that mTORC1 positively regulates VEGF expression and secretion in osteoblasts, which did not account for the corresponding angiogenesis alterations in mutant mouse bone and *in vitro*, suggesting that osteoblasts may produce angiostatic factor(s) to block VEGF signalling in bone.

### Cxcl9 is constitutively produced by osteoblasts

**Figure 4:**
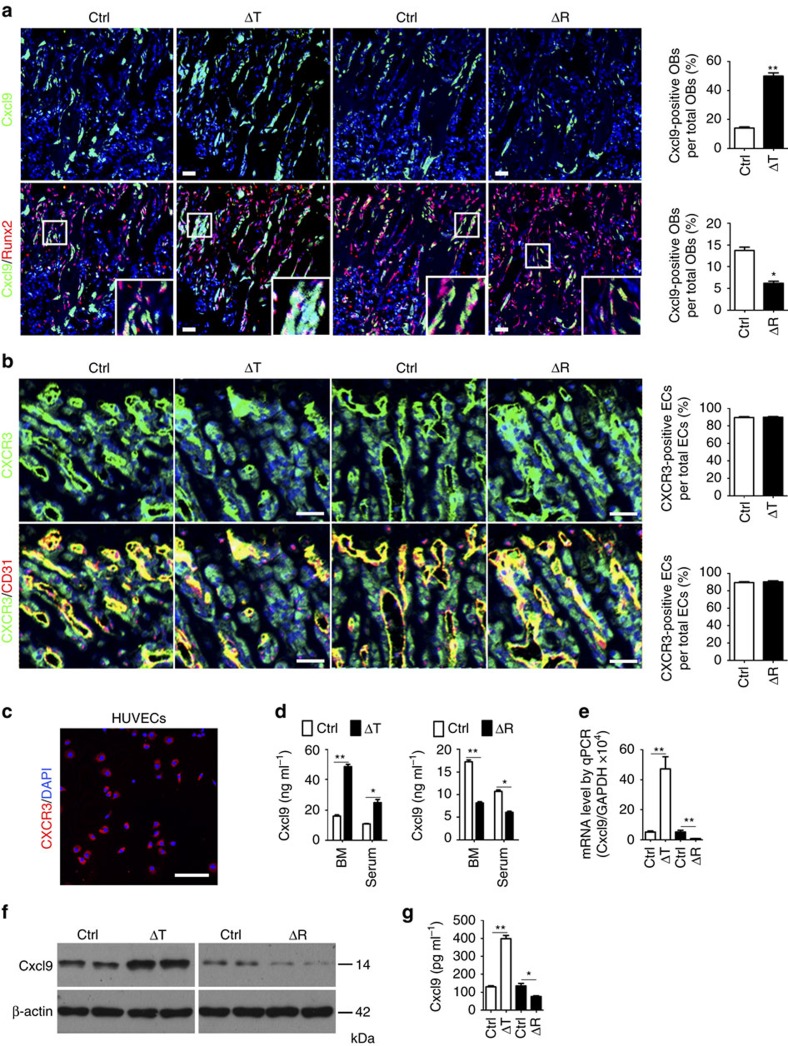
mTORC1 regulates Cxcl9 in osteoblasts. (**a**) Representative images of *in situ* hybridization of Cxcl9 mRNA in conjunction with immunostaining of Runx2 in femur sections of 12-week-old male mice bone. Boxed area is enlarged in the bottom right corner. Cxcl9^+^ osteoblasts out of total osteoblasts were also quantified. Scale bar, 50 μm. *n*=9 per group. (**b**) Representative photomicrographs of immunostaining of CXCR3 in CD31^+^ ECs in bone marrow and quantitative analysis of CXCR3^+^ ECs out of total ECs in 12-week-old male mice bone. Scale bar, 50 μm. *n*=9 per group. (**c**) Representative photomicrographs of immunostaining of CXCR3 in cultured HUVECs. Scale bar, 100 μm. (**d**) Cxcl9 concentrations assessed by ELISA in bone marrow (BM) and serum. *n*=5 per group. (**e**) Quantitative PCR analysis of Cxcl9 mRNA in primary osteoblasts. (**f**) Western blot of Cxcl9 in primary osteoblasts. (**g**) Concentrations of Cxcl9 in CM of primary osteoblasts assessed by ELISA. *n*=5 per group. Data are shown as mean±s.d. *P<0.05, **P<0.01 (Student's *t*-test). Ctrl, control.

To screen for the angiostatic factor(s), we developed a global mRNA expression profile in Δ*Tsc1* or control calvarial osteoblasts using microarray. Among the upregulated mRNAs, Cxcl9 has been reported as a direct counter-regulatory molecule of VEGF signalling within the liver^26^. Importantly, Cxcl9 was found to be expressed constitutively in osteoblasts in cells residing in the bone (Fig. 4a), and its receptor, CXCR3, was revealed to be expressed by ECs in bone marrow and by cultured HUVECs (Fig. 4b,c). CXCR3 presented consistent expression in the two transgenic mouse models and their respective controls (Fig. 4b), but Cxcl9 was expressed and secreted into bone marrow and serum at significantly greater levels by Δ*Tsc1* osteoblasts (Fig. 4a,d). Furthermore, Cxcl9 mRNA expression showed a sevenfold increase in primary Δ*Tsc1* osteoblasts versus control cells (Fig. 4e). A similar increased pattern was further confirmed at the protein expression (Fig. 4f) and secretion level (Fig. 4g).

In contrast to Δ*Tsc1* mice, Δ*Raptor* osteoblasts had significantly lower levels of Cxcl9 expression (Fig. 4a) and secretion (Fig. 4d). Cxcl9 mRNA (Fig. 4e), protein expression (Fig. 4f) and secretion (Fig. 4g) were all decreased in Δ*Raptor* calvarial osteoblasts cultured *in vitro*. On the basis of these observations, we conclude that mTORC1 positively regulates Cxcl9 expression and secretion in osteoblasts, indicating a possible explanation for the angiogenesis phenotypes of mutant mice described above.

### Cxcl9 inhibits angiogenesis in bone

**Figure 5:**
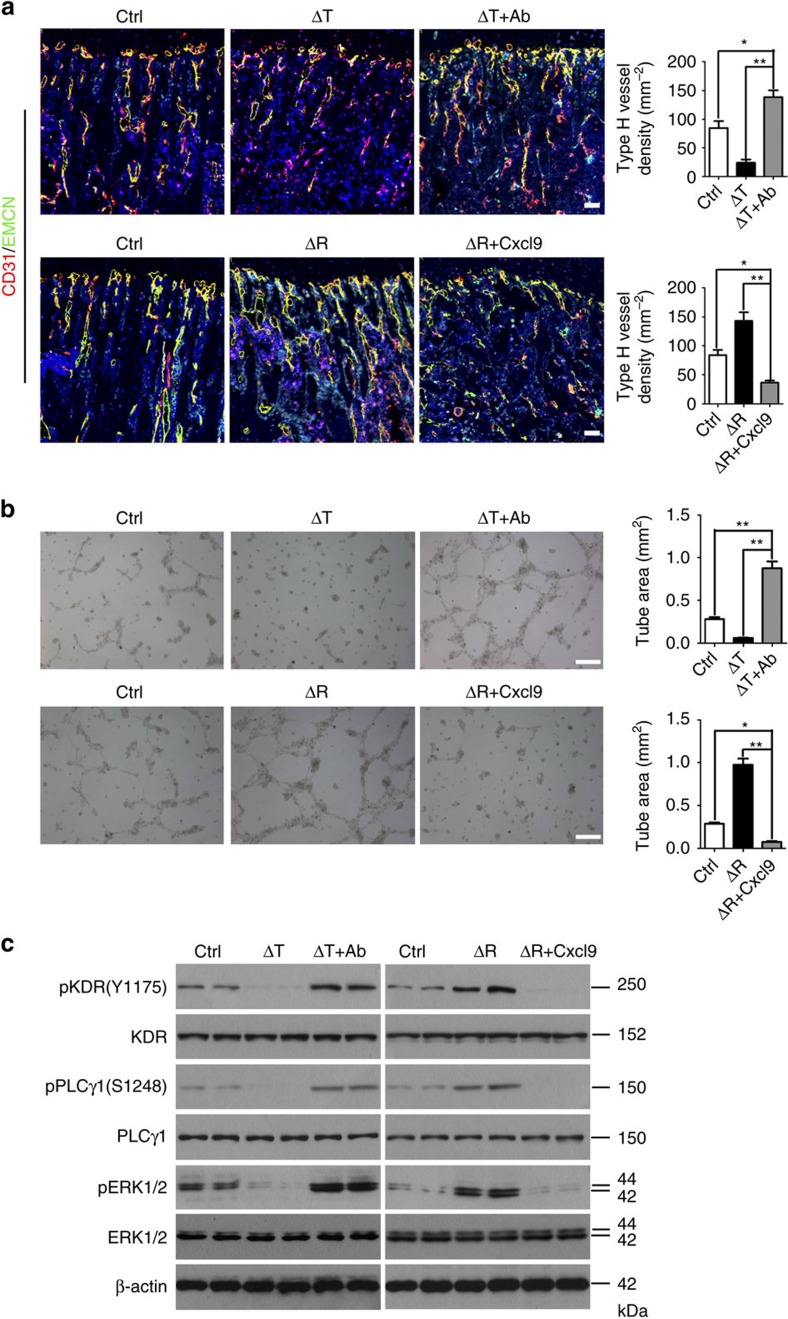
Cxcl9 is responsible for vascular phenotypes in mutant mouse bone. (**a**) Representative confocal images of microvessels immunostained by CD31 and EMCN, and quantitative analysis of microvessel density in tibia sections of Δ*Tsc1* mice administered with Cxcl9 antibody (Ab) and Δ*Raptor* mice injected with Cxcl9 subcutaneously. Scale bar, 100 μm. *n*=9 per group. (**b**) Representative Matrigel tube formation assay images and quantitative analysis of tube area with cultures of HUVECs using CM with or without addition of Cxcl9 or Cxcl9-neutralizing antibody as indicated. Scale bar, 100 μm. *n*=9 per group. (**c**) Western blot of phosphorylation of KDR, PLCγ1 and ERK1/2 in HUVECs treated with CM from primary osteoblasts with or without addition of Cxcl9 or Cxcl9-neutralizing antibody as indicated for 10 min. Data are shown as mean±s.d. *P<0.05, **P<0.01 (Student's *t*-test). Ctrl, control.

We next sought to determine whether Cxcl9 is responsible for the vasculature alterations seen in the two mouse models. Δ*Tsc1* mice were treated with anti-Cxcl9 antibody to neutralize endogenous Cxcl9. Interestingly, antibody against Cxcl9 significantly increased the number of bone CD31^+^Endomucin^+^ vessels in Δ*Tsc1* mice to more than that in control mice (Fig. 5a). As depicted above, HUVECs maintained in Δ*Tsc1* CM exhibited impaired proliferation and migration. When the cells were grown in the same medium supplemented with anti-Cxcl9, proliferation (Supplementary Fig. 4a) and migration rates (Supplementary Fig. 4b) of the cells were significantly elevated to levels higher than cells treated with the control medium. Moreover, an endothelial network was formed when HUVECs were grown in Δ*Tsc1* CM with supplementary anti-Cxcl9, compared with those cultured in CM from Δ*Tsc1* osteoblasts and control osteoblasts (Fig. 5b). Importantly, reduced phosphorylation of KDR and its downstream mediator in HUVECs were both increased to above basal level (Fig. 5c). In addition, Cxcl9 was downregulated in Δ*Tsc1* osteoblasts by siRNA, CM from which also promoted the proliferation, migration and tube formation of HUVECs (Supplementary Fig. 5). These data suggested that elevated Cxcl9 in osteoblasts is responsible for the reduced vasculature in bone of Δ*Tsc1* mice. As the angiostatic effect of Cxcl9 was eliminated, transduction of VEGF signalling recovered and the abundant VEGF expressed in Δ*Tsc1* osteoblasts exerted their potential effect on promoting angiogenesis in bone and *in vitro*.

**Figure 6:**
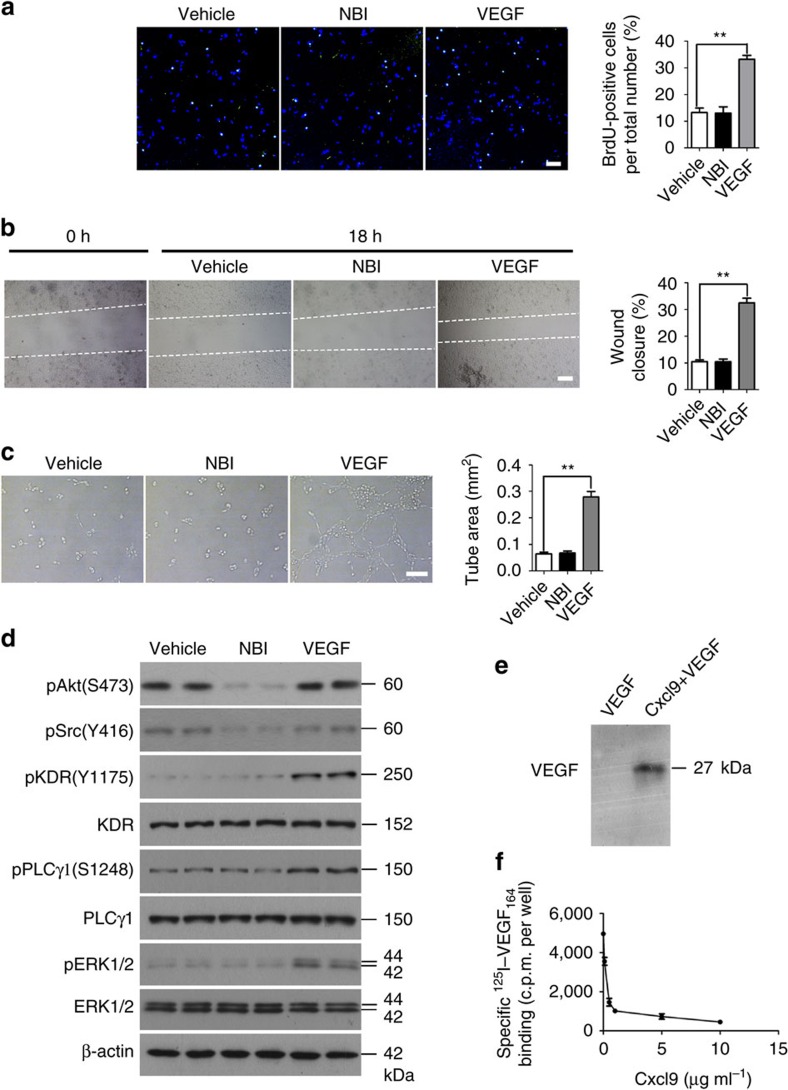
Cxcl9 antagonizes VEGF signalling transduction in ECs by interacting with VEGF and preventing its binding to ECs. (**a**) Representative confocal images of immunostaining of BrdU (green) in HUVECs and quantitative analysis of BrdU^+^ cells over total cells. Scale bar, 100 μm. *n*=9 per group. (**b**) Representative photomicrographs of wounds in HUVECs at 0 h and after 18 h; dotted lines highlight the linear scratch/wound for each group of cells. The bar graph shows the mean percentage of wound closure. Scale bar, 200 μm. *n*=9 per group. (**c**) Representative photomicrographs of tube formation of HUVECs incubated with Matrigel and quantitative analysis of tube area. Scale bar, 200 μm. *n*=9 per group. (**d**) Western blot of phosphorylation of Akt (S473), Src (Y416), KDR, PLCγ1 and ERK1/2 in HUVECs treated with Δ*Tsc1* CM with or without addition of NBI-74330 (CXCR3 antagonist) or VEGF as indicated for 10 min. (**e**) Recombinant mouse Cxcl9 and VEGF_164_ were mixed and immunoprecipitated with anti-Cxcl9 antibody and examined by immunoblotting with an anti-VEGF antibody. (**f**) Binding of ^125^I–VEGF_164_ to ECs in the presence of increasing concentrations of Cxcl9. Shown is the specific binding, which was calculated by subtracting the nonspecific binding from the total binding. Data are shown as mean±s.d. **P<0.01 (Student's *t*-test).

To test whether reduced Cxcl9 in Δ*Raptor* osteoblasts contributed to increased vasculature in bone, we treated Δ*Raptor* mice with Cxcl9. Δ*Raptor* mice receiving Cxcl9 had significantly fewer vessels than those treated with phosphate-buffered saline (PBS) and control mice (Fig. 5a). Cxcl9 also significantly reversed the promotion of proliferation and migration by Δ*Raptor* CM in HUVECs and further reduced proliferation (Supplementary Fig. 6a) and migration (Supplementary Fig. 6b) to rates lower than those in cells maintained in control medium. In the Matrigel assay, the network in HUVECs maintained in Δ*Raptor* medium supplemented with Cxcl9 was less formed than that grown in control medium (Fig. 5b). Immunoblotting analysis of lysates of the cell sets revealed the underlying mechanism. As transduction of VEGF signalling was improved in HUVECs maintained in Δ*Raptor* CM, signal transduction in cells was blocked by supplementation of Cxcl9 in the same medium (Fig. 5c). These observations indicated that decreased Cxcl9 expression in osteoblasts was responsible for the increased vasculature in bone of Δ*Raptor* mice. As Cxcl9 was downregulated in Δ*Raptor* osteoblasts, blocking VEGF signal transduction by Cxcl9 was alleviated in ECs, and VEGF released by Δ*Raptor* osteoblasts was able to exert its pro-angiogenic role more effectively and led to the formation of more vessels in the bone. Together, these findings indicate that Cxcl9 antagonizes VEGF signalling to prevent angiogenesis in bone. We next determined the mechanisms by which Cxcl9 antagonizes VEGF signalling transduction in ECs. We first examined whether CXCR3 (Cxcl9 receptor) mediated the antiangiogenic effects of Cxcl9 in our model. As CXCR3 was abrogated by its antagonist NBI-74330 supplemented in Δ*Tsc1* CM, HUVECs cultured in the CM retained a low rate of proliferation (Fig. 6a) and migration (Fig. 6b), and a poor capacity to form tube (Fig. 6c). Furthermore, NBI-74330 blocked CXCR3 activity but failed to alleviate the inhibition of VEGF signalling transduction in ECs by Cxcl9 in Δ*Tsc1* CM (Fig. 6d), indicating that CXCR3 is not required for Cxcl9 to suppress angiogenesis in this model. On the other hand, supplementation with VEGF markedly reversed the inhibition of angiogenesis by Δ*Tsc1* CM (Fig. 6a–d). In light of these observations, we hypothesized that Cxcl9 could interact with VEGF and attenuate its binding to ECs. We further mixed recombinant mouse Cxcl9 and VEGF_164_ *in vitro* and immunoprecipitated VEGF using an anti-Cxcl9 antibody. As shown in Fig. 6e, the anti-Cxcl9 antibody successfully precipitated VEGF_164_ from the mixture of VEGF_164_ and Cxcl9, but failed to precipitate VEGF_164_ from the solution containing VEGF_164_ alone. These results suggest that Cxcl9 interacts with VEGF_164_ *in vitro*. To investigate the effect of Cxcl9 on the binding of VEGF to ECs, HUVECs were incubated with 10 ng ml^−1 125^I–VEGF_164_ and increasing concentrations of Cxcl9. As expected, Cxcl9 was able to inhibit the specific binding of ^125^I–VEGF_164_ to ECs (Fig. 6f). Together, these findings revealed that Cxcl9 interacts with VEGF and prevents VEGF from binding to ECs, and thus antagonizes VEGF signal transduction in ECs.

### Cxcl9 suppresses osteogenesis

**Figure 7:**
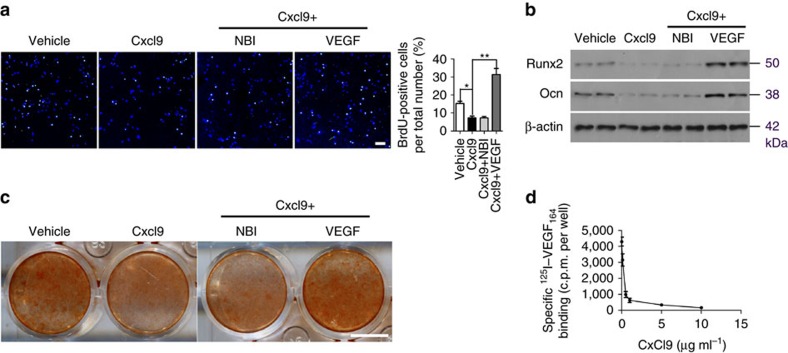
Cxcl9 suppresses osteogenesis by interacting with VEGF and abrogating binding of VEGF to osteoblasts. (**a**) BrdU staining of MC3T3-E1 cells and quantitative analysis of BrdU^+^ cells out of total cells. Scale bar, 100 μm. (**b**) Western blot analysis of osteoblastic marker Ocn and Runx2 expression in MC3T3-E1 cells on the seventh day of osteogenic induction. (**c**) Alizarin red staining of differentiated MC3T3-E1 cells on the fourteenth day. Scale bar, 1 cm. (**d**) Binding of ^125^I–VEGF_164_ to MC3T3-E1 cells in the presence of increasing concentrations of Cxcl9. Shown is the specific binding, which was calculated by subtracting the nonspecific binding from the total binding. Data are shown as mean±s.d. *P<0.05, **P<0.01 (Student's *t*-test).

Given that angiogenesis and osteogenesis are coupled in bone, we next investigated whether Cxcl9 has any direct role in bone formation. After treatment with the Cxcl9 antibody depicted above, Δ*Tsc1* mice exhibited partial recovery of osteoblastic differentiation, mineralization of bone matrix and normalization of bone structure (Supplementary Fig. 1). In contrast, Δ*Raptor* mice receiving Cxcl9 showed impairment of osteoblastic differentiation and more severe loss of bone mass (Supplementary Fig. 2). As CXCR3 expression in osteoblasts is consistent between the two knockout mice and their controls (Supplementary Fig. 7a), we suspected that Cxcl9 may exert inhibitory effect on the function of osteoblasts. To better characterize the role of Cxcl9 in osteoblasts, we added recombinant Cxcl9 to cultured MC3T3-E1 cells, had CXCR3 expression (Supplementary Fig. 7b). Cxcl9 demonstrated a marked inhibitory effect on proliferation (Fig. 7a), differentiation (Fig. 7b) and mineralization of MC3T3-E1 cells (Fig. 7c). Since inhibition of CXCR3 by NBI-74330 failed to reverse the abrogation of osteogenesis by Cxcl9 (Fig. 7a–c), we suspected that Cxcl9 may inhibit osteogenesis through a CXCR3-independent mechanism. Interestingly, supplementation with VEGF significantly reversed the inhibitory effect of Cxcl9 on the osteogenesis of MC3T3-E1 cells (Fig. 7a–c), indicating that Cxcl9 may prevent osteogenesis by interacting with VEGF. Indeed, Cxcl9 was able to abrogate the binding of VEGF to MC3T3-E1 cells *in vitro* (Fig. 7d). Furthermore, Cxcl9 inhibited proliferation, differentiation and mineralization of rat bone marrow stem cells (BMSCs) and prevented binding of VEGF to these cells as well (Supplementary Fig. 8). Taken together, these observations indicate that Cxcl9 may suppress osteogenesis in bone and *in vitro* by interacting with VEGF and abrogating binding of VEGF to osteoblasts.

### mTORC1 regulates Cxcl9 in osteoblasts via STAT1

**Figure 8:**
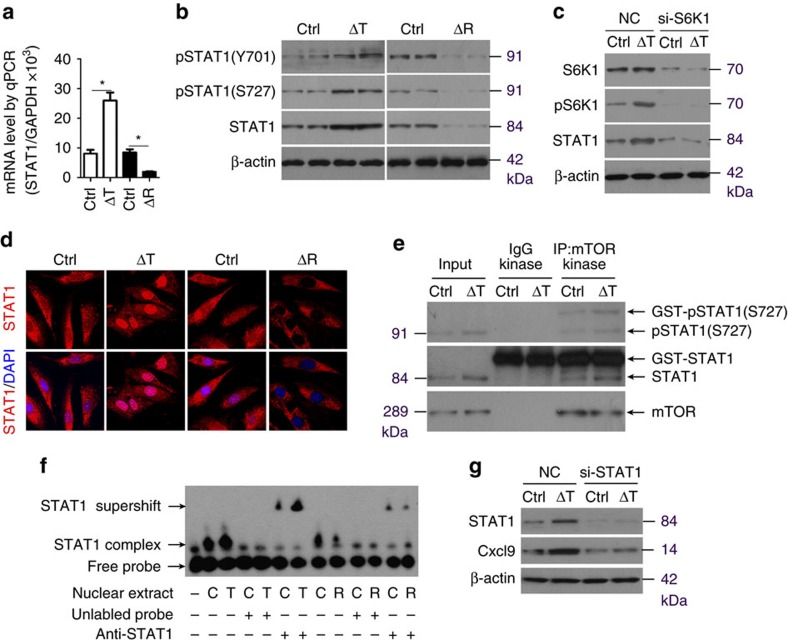
mTORC1 regulates CXCL9 in osteoblasts via STAT1. (**a**) Quantitative PCR (qPCR) analysis of STAT1 mRNA in primary osteoblasts. *n*=3 per group. Data are shown as mean±s.d. *P<0.05 (Student's *t*-test). (**b**) Western blot of total STAT1 protein and phosphorylation (Y701 and S727) of STAT1 in primary osteoblasts. (**c**) Representative confocal images show subcellular location of STAT1 (red) in primary osteoblasts. (**d**) Control and Δ*Tsc1* (ΔT) primary osteoblasts were treated with S6K1 siRNA and negative control (NC) for 48 h and then immunoblotting was carried out to detect STAT1 expression. (**e**) Cultured primary calvarial cells were immunoprecipitated with anti-mTOR antibody and the precipitated mTOR was assayed for kinase activity against recombinant glutathione *S*-transferase (GST)-tagged full-length STAT1. (**f**) Nuclear extracts from primary Δ*Tsc1* (T), Δ*Raptor* (R) and control (C) osteoblasts were analysed for binding of STAT1 to *Cxcl9* promoter using EMSA. Binding of STAT1 to biotin-labelled DNA probes is shown as ‘STAT1 complex'. To compete with the binding, an unlabelled STAT1-binding-site DNA probe was added to the reaction in 200 times molar excess. Adding anti-STAT1 antibody to the reactions caused a reduction of STAT1-DNA binding and bands of supershift. (**g**) Control and Δ*Tsc1* primary osteoblasts were treated with STAT1 siRNA and NC for 48 h and then immunoblotting was carried out to detect Cxcl9 expression. Ctrl, control.

We next investigated the mechanism by which mTORC1 regulates Cxcl9 in osteoblasts. The transcription factor, signal transducer and activator of transcription 1 (STAT1), is known to be regulated by mTOR^27^,^28^ and is involved in the regulation of *Cxcl9* gene transcription^29^ in many cell types. We thus examined the potential regulation of STAT1 by mTORC1 in osteoblasts and found that RNA transcripts for *STAT1* were upregulated in Δ*Tsc1* calvarial osteoblasts and were significantly reduced in Δ*Raptor* cells (Fig. 8a). Accordingly, STAT1 protein production and phosphorylation was revealed to be increased in Δ*Tsc1* and decreased in Δ*Raptor* osteoblasts (Fig. 8b). The function of many transcription factors is associated with changes in their intracellular localization between the cytoplasm and the nucleus. We then found that STAT1 was more concentrated in the nucleus of Δ*Tsc1* osteoblasts (Fig. 8c). In contrast, Δ*Raptor* osteoblasts showed STAT1 accumulation in the cytoplasm (Fig. 8c). These findings suggested that mTORC1 promoted STAT1 expression and translocation into the nucleus in osteoblasts.

We next investigated the mechanisms by which mTORC1 drives STAT1 expression and activation. To assess the involvement of S6K1 in the regulation of STAT1 expression by mTORC1, we downregulated S6K1 in primary calvarial osteoblasts using siRNA. S6K1 reduction led to decreased STAT1 in control osteoblasts and reversed the upregulation of STAT1 expression in Δ*Tsc1* osteoblasts (Fig. 8d), suggesting that S6K1 mediated the positive regulation of STAT1 expression by mTORC1 in osteoblasts. STAT1 phosphorylated on serine 727 enhances its transcriptional activity^30^. As shown in Fig. 8e, mTOR immunoprecipitates phosphorylate STAT1 at Ser727 *in vitro* and constitutive activated mTORC1 from Δ*Tsc1* calvarial cells enhanced phosphorylation, suggesting that mTOR directly phosphorylates STAT1 at Ser727 and promotes its transcriptional activity.

We also performed electrophoretic mobility shift assay (EMSA) to delineate STAT1 binding to the *Cxcl9* gene promoter. Nuclear protein of osteoblasts bound specifically to an oligonucleotide probe containing a consensus STAT1-specific binding sequence in the promoter region of *Cxcl9* (Fig. 8f). The addition of antibody to STAT1 in the nuclear extract displaced the binding band (Fig. 8f), indicating that this binding complex contained STAT1. Importantly, nuclear protein from Δ*Tsc1* osteoblasts showed elevated binding of STAT1 to the probe, while Δ*Raptor* nuclear protein showed decreased binding (Fig. 8f). These observations indicated that STAT1 was bound to the *Cxcl9* gene promoter in osteoblasts, and mTORC1 promoted this binding.

The role of STAT1 in mediating mTORC1-regulated expression of Cxcl9 was finally determined by siRNA knockdown of STAT1 mRNA in osteoblasts. In control cells, the basal level of Cxcl9 was decreased significantly by STAT1 siRNA (Fig. 8g). Moreover, si-STAT1 markedly reversed the upregulation of Cxcl9 by mTORC1 activation in Δ*Tsc1* cells (Fig. 8g). These results confirmed that STAT1 is involved in the regulation of Cxcl9 gene transcription by mTORC1 in osteoblasts.

## Discussion

**Figure 9:**
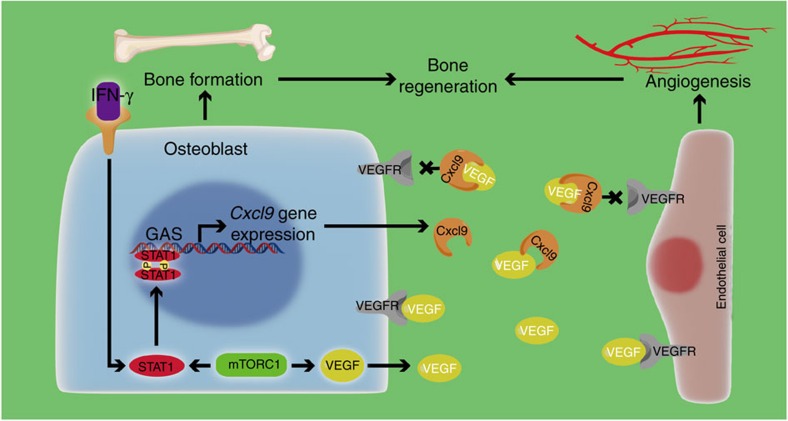
Model of Cxcl9 secreted by osteoblasts in regulating angiogenesis and osteogenesis in bone. Cxcl9 expression is positively regulated by mTORC1 and downstream STAT1 in osteoblasts. Cxcl9 binds with VEGF, prevents VEGF from binding to its receptors, blocks VEGF signalling transduction in ECs and thus inhibits angiogenesis in bone. In addition, Cxcl9 suppresses osteogenesis by interacting with VEGF and abrogating its binding to osteoblasts. IFN-γ, interferon-gamma.

Using mouse models with mTORC1 activation or inhibition in osteoblasts, we identified Cxcl9 as an essential factor secreted by osteoblasts, which modulate angiogenesis and osteogenesis in bone. We found that Cxcl9 was expressed constituently in osteoblasts in cells residing in the bone, and its expression was positively regulated by mTORC1 upstream of STAT1. Cxcl9 inhibited blood vessel formation by interacting with VEGF and preventing its binding to ECs, which was sufficient to reverse the angiogenic effect of VEGF. Cxcl9 suppressed osteogenesis in bone and *in vitro* by antagonizing VEGF as well. Thus, Cxcl9 is a novel angiostatic factor that mediates the communication between osteoblasts and ECs during bone regeneration (Fig. 9).

Bone regeneration occurs in close spatial and temporal association with angiogenesis, thus the rate of bone formation and blood flow in the animal are usually coupled^31^. However, our transgenic mouse models showed the contrary. The Δ*Tsc1* mice exhibited increased bone volume but low vascularization, while the Δ*Raptor* mice had low bone mass but increased vascularization. These inconsistencies might reveal a protective mechanism in these mice to maintain appropriate bone mass. Hyperactive mTORC1 in osteoblasts resulted in excess bone mass in Δ*Tsc1* mice (Supplementary Fig. 1). In this extreme situation, reduction of vascularization was beneficial for deceasing bone mass. In contrast, as *Raptor* deficiency in osteoblasts induced low bone volume (Supplementary Fig. 2), regaining bone mass requires abundant blood flow in Δ*Raptor* mice. Despite no coupling between angiogenesis and osteogenesis was observed, cross-talk between bone-forming osteoblasts and ECs mediated by Cxcl9 was witnessed in these genetic models. Moreover, as Cxcl9 exerts dual inhibitory role in angiogenesis and osteogenesis, both of which are essential for maintaining bone mass, Cxcl9 inhibition is a promising therapeutic strategy for bone loss diseases.

Activation of Cxcl9 expression has been reported in several stress-related situations, including malignancies^32^, chemotherapy^33^, graft-versus-host diseases^34^ and infections^35^,^36^. For the first time, we demonstrated that Cxcl9 is expressed constitutively in osteoblasts. In contrast, Lisignoli *et al*.^37^ found that Cxcl9 was undetectable in cultured human osteoblasts^37^. We speculate that these different results may have arisen due to different cell types and sensibility of the detection methods for Cxcl9. We failed to detect Cxcl9 protein in osteoblasts in bone when performing an antibody-dependent immunohistochemical assay. However, *in situ* hybridization revealed marked Cxcl9 mRNA expression in osteoblasts. As a secretory protein, the majority of Cxcl9 is secreted in extracellular fluid after synthesis from mRNA, leaving a small quantity in the cell below detection. High-level secretion provides preconditions for Cxcl9 to exert its role in the microenvironment of bone marrow.

Cxcl9 generally exerts its effect by binding with its receptor CXCR3 (refs 38, 39); however, our data showed that Cxcl9 abrogate angiogenesis and osteogenesis independent of CXCR3. Instead, Cxcl9 interacts with VEGF and prevents its binding to ECs and osteoblasts. Analogously, PF4, another CXC-chemokine as well as CXCR3 ligand, has also been reported to be able to bind VEGF and inhibit its receptor-binding ability^40^. On the other hand, as immunohistochemical staining of CXCR3 revealed its expression in other cells besides ECs and osteoblasts residing in bone marrow, we could not rule out that an indirect effect of Cxcl9 on these cells would contribute to the vascular and bone phenotypes observed in our mouse model. However, ECs and osteoblasts cultured *in vitro* were subjected to the same effects as those in bone marrow when exposed directly to Cxcl9, which consolidated our conclusion that Cxcl9 is responsible for the vascular and bone phenotypes in mouse bone. Moreover, Cxcl9 may exert its other biological effects in addition to inhibiting angiogenesis and osteogenesis on other cell types. Thus, other potential alterations in bone in our mouse model may have occurred, which are outside the scope of this study.

Our additional data showed that the number of osteoblasts per bone perimeter was increased in Δ*Tsc1*mice but decreased in Δ*Raptor* mice (Supplementary Fig. 9), which may have partially contributed to the respective alteration in Cxcl9 secretion in bone marrow and sera in these two mouse models. However, as Cxcl9 mRNA expression, calibrated by GAPDH, was revealed to be elevated in the primary Δ*Tsc1* osteoblasts and decreased in Δ*Raptor* osteoblasts, Cxcl9 expression in single cells should be increased in Δ*Tsc1* osteoblasts and reduced in Δ*Raptor* osteoblasts. This evidence shows that mTORC1 positively regulates Cxcl9 expression in osteoblasts. Mechanistically, we show that mTORC1 regulates Cxcl9 in osteoblasts by modulating STAT1 expression and activity. Cxcl9 expression is determined largely by the control of STAT1 nuclear content and binding of STAT1 to *Cxcl9* gene promoters^29^. A considerable quantity of STAT1 was shown to be present in the nucleus of control osteoblasts (Fig. 8c), explaining the constituent expression of Cxcl9 in osteoblasts. Moreover, STAT1 mRNA and protein were increased in osteoblasts with activated mTORC1 and were decreased in those with impaired mTORC1, which demonstrated positive transcriptional regulation of STAT1 by mTORC1 in osteoblasts. In support of these observations, EI-Hashemite *et al*.^27^ demonstrated a marked increase in STAT1 expression in tumours and mouse embryo fibroblast cell lines that lacked either *Tsc1* or *Tsc2* (ref. 27).

In summary, our study identified Cxcl9 as an angiostatic factor secreted by osteoblasts to regulate angiogenesis and osteogenesis in bone and revealed mTORC1 signalling and STAT1 as critical upstream mediators. Pharmaceutical coordination of the pathways and agents may be beneficial in bone formation.

## Methods

### Mice

We purchased the *Tsc1*^*flox/flox*^*, Raptor*^*flox/flox*^ and *OC-Cre* mouse strains from Jackson Laboratory. The background of *Tsc1*^*flox/flox*^ mice is 129S4/SvJae, and these mice were backcrossed to mice with a C57BL/6 background for eight generations before use. We performed genotyping using genomic DNA isolated from tail biopsies, and the primers used were as follows: loxP *Tsc1* allele forward, 5′-GTCACGACCGTAGGAGAAGC-3′ and reverse, 5′-GAATCAACCCCACAGAGCAT-3′; loxP *Raptor* allele forward, 5′-CTCAGTAGTGGTATGTGCTCAG-3′ and reverse, 5′-GGGTACAGTATGTCAGCACAG-3′; *OC-Cre* forward, 5′-CAAATAGCCCTGGCAGATTC-3′ and reverse, 5′-TGATACAAGGGACATCTTCC-3′.

The mice (male, 4-week-old, three mice per each group) were subcutaneously administered mouse CXCL9 antibody (R&D System, #AF-492-NA, 1 μg per 50 μl) or rMuMig/CXCL9 (PrimeGene Bio-Tech, #221-09, 100 ng per 50 μl) every other day for 2 months.

Mice importation, transportation, housing and breeding were conducted according to the recommendations of ‘The use of non-human primates in research.' The mice were killed by cervical dislocation to prevent suffering. The Southern Medical University Animal Care and Use Committee approved all procedures involving the mice.

### Micro-CT analysis

Femora were dissected from 12-week-old male mice (five mice per each group), fixed for 48 h in 4% paraformaldehyde and analysed at 12 μm resolution on a micro-CT Scanner (Viva CT40; Scanco Medical AG, Bassersdorf, Switzerland). In brief, we scanned the lower growth plate in the femora and extended proximally for 300 slices. We started morphometric analysis with the first slice in which the femoral condyles were fully merged and extended for 100 slices proximally. Using a contouring tool, we segmented the trabecular bone from the cortical shell manually on key slices, and morphed the contours automatically to segment the trabecular bone on all slices. The three-dimensional structure and morphometry were constructed and analysed for trabecular bone volume fraction (BV/TV), trabecular thickness (Tb. Th), trabecular number (Tb. N) and trabecular separation (Tb. Sp). We also performed micro-CT imaging in the mid-diaphysis of the femur and performed midshaft evaluation of 100 slices to quantify the cortical thickness (Ct. Th) and periosteal perimeter (Ps. Pm).

### Immunostaining of slices and cells and histomorphometric analyses

Hindlimb tissues from the mice were fixed using 4% paraformaldehyde in PBS at 4 °C for 48 h and then decalcified in 15% ethylenediaminetetraacetic acid (EDTA; pH 7.4) at 4 °C for 14 days. The tissues were embedded in paraffin or optimal cutting temperature compound (Sakura Finetek), and 2–5 μm sagittal-oriented sections were prepared for histological analyses. For immunohistochemistry, we incubated primary antibodies that recognized mouse phospho-S6 (Ser235/236) (Cell Signaling, #2211, 1:100), osteocalcin (Abcam, #ab93876, 1:500), CD31 (Abcam, #ab28364, 1:50), VEGF (Proteintech Group, #19003-1-AP, 1:200),phospho-VEGFR2 (Y1175) (Cell Signaling, #2478, 1:100) and CXCR3 (Santa Cruz Biotechnology, #sc-13951,1:50) overnight at 4 °C. Subsequently, we used secondary antibodies conjugated with fluorescence at room temperature for 1 h. We counted the numbers of positively stained cells or vessels (CD31^+^) in the whole diaphyseal periosteum or four random visual fields of metaphysis per femur or tibia in three sequential sections per mouse in each group. We calculated VEGF^+^ osteoblasts/total osteoblasts (%) as the numbers of cells double-stained with VEGF and osteocalcin compared with osteocalcin-positive cells, and determined microvessel density as the number of CD31 (and endomucin (EMCN))-positive vessels per area of bone marrow. *In situ* hybridization with digoxin-labelled probes was performed using a Cxcl9 mRNA *in situ* hybridization kit (Boster, #MK3237). We used FluoView FV1000 confocal microscopy (Olympus) or an Olympus BX51 microscope for imaging samples.

For immunocytochemical staining, we incubated cultured cells with primary antibody to mouse STAT1 (Proteintech, #10144-2-AP, 1:100) overnight at 4 °C. Subsequently, we used secondary antibodies conjugated with fluorescence at room temperature for 1 h while avoiding light. FluoView FV1000 confocal microscopy (Olympus) was used for imaging.

### Cells

Primary osteoblastic cells were prepared from the calvaria of newborn mice. In brief, calvariae were dissected from the mice (24 h after birth), rinsed with PBS and digested in freshly made 0.1 mg ml^−1^ collagenase type II (Thermo Fisher Scientific, #17101015) in α-minimal essential Eagle's medium at 37 °C for 20 min; the digestion was repeated five times. After digestion, supernatants were combined and centrifuged to pellet cells^41^,^42^. Cells were then maintained in α-MEM (Gibco) supplemented with 10% fetal bovine serum (Gibco), 100 U ml^−1^ penicillin and 100 mg ml^−1^ streptomycin sulfate, at 37 °C with 5% CO2. After reaching confluence in 60 mm culture dishes, the medium was replaced with α-MEM (Gibco) supplemented with 1% bovine serum albumin, and the cells were cultured for 16 h before collecting the CM. Mouse CXCL9 antibody (R&D System, #AF-492-NA, 2 μg ml^−1^), rMuMig/CXCL9 (PrimeGene Bio-Tech, #221-09, 250 ng ml^−1^), CXCR3 antagonist NBI-7433(R&D System, #4528, 1 μM) or mouse recombinant VEGF_164_ (R&D System, #493-MV-025, 10 ng ml^−1^) was added to the CM as indicated.

HUVECs were purchased from the American Type Culture Collection and were cultured in EC basal medium 2 (EBM2) containing low serum (2% fetal calf serum) and EC growth supplement (Promo Cell). Cells were serum-deprived for 16 h before CM was added.

### Cell proliferation assay

HUVECs were serum-starved in EBM2 medium (0.1% fetal bovine serum without growth factor; Lonza, USA) for 16 h, and then seeded (200 μl containing 8,000 cells per well) into the hole of a confocal dish (Bioimager, #100350) and incubated for 4 h. The cells were then treated with BrdU labelling reagent (Invitrogen) for 2 h according to the manufacturer's instructions and washed with PBS. The cells were fixed with 70% ethanol for 25 min at room temperature, and then stained for immunocytochemical analysis. Nine areas in each group were counted by two independent observers blinded to the groups. We scored BrdU-positive cells over total cells visually and with Image Pro Plus software.

### *In vitro* migration assays

HUVECs were seeded (1 × 10^5^ cells per well) in 1% gelatin-coated 24-well plates (Corning, Schiphol, Netherlands). Confluent cells were serum-deprived for 16 h, and a linear wound was created in monolayers by scratching with a sterile pipette tip (200 μl yellow tip). Monolayers were washed with PBS to remove floating cells and the CM was added. After an additional 18 h, cell migration into the wound was assessed by microscopy using a digital inverted microscope. The degree of wound closure was measured as the percentage of the area covered by migrating cells in the initial wound in nine wounds per test condition, using Image Pro Plus software.

### *In vitro* tube formation assay

HUVECs were serum-starved for 16 h and then seeded at a density of 10,000 per well on growth factor-depleted Matrigel (BD Biosciences, NSW, Australia) in 24-well plates. CM was added, and the results were quantified 6 h later. Microscopic fields containing the tube structures formed in the gel were photographed at low magnification ( × 10). Nine fields per test condition were examined. Before they were photographed, the cells were fixed with 4% paraformaldehyde. Tube area was quantified using Image Pro Plus software.

### Collection of bone marrow supernatant

Two-month-old male mice (five per each group) were killed and the bone marrow was exposed by cutting two ends of the tibia. Samples were then centrifuged for 15 min at 3,000 r.p.m. and 4 °C to obtain bone marrow supernatants, which were then stored at −80 °C until ELISA analysis.

### ELISA analysis

We used the Mouse VEGF ELISA Kit (Elabscience Biotechnology, #E-EL-M0050) and Mouse CXCL9/MIG (Monokine induced by interferon-gamma) ELISA Kit (Elabscience, # E-EL-M0020) to analyse VEGF and Cxcl9 in serum, bone marrow supernatant and CM, respectively. We performed the ELISA analysis according to the manufacturers' instructions.

### Western blot

We lysed cells with 2% SDS, 2 M urea, 10% glycerol, 10 mM Tris-HCl (pH 6.8), 10 mM dithiothreitol and 1 mM phenylmethylsulfonyl fluoride. The lysates were centrifuged and the supernatants were separated by SDS–polyacrylamide gel electrophoresis and blotted onto a nitrocellulose membrane (Bio-Rad Laboratories). The membrane was then incubated with specific antibodies to phospho-S6K (T389) (Cell Signaling Technology, #9234, 1:1,000), S6K (Santa Cruz Biotechnology, #sc-8418, 1:2,000), phospho-S6 (S235/236) (Cell Signaling Technology, #2211, 1:1,000), S6 (Santa Cruz Biotechnology, #sc-74459, 1:2,000), VEGF (Proteintech Group, #19003-1-AP, 1:1,000), phospho-VEGFR2 (Y1,175) (Abclonal Technology, #AP0382, 1:1,000), VEGFR2 (Abclonal Technology, #A7695, 1:1,000), phospho-PLCγ1(S1,248) (Cell Signaling Technology, #8713, 1:1,000), PLCγ1 (Abclonal Technology, #A7711, 1:1,000), phospho-ERK1/2 (Thr202/Tyr204) (Cell Signaling Technology, #4370, 1:1,000), ERK1/2 (Abclonal Technology, #A0229, 1:1,000), phospho-Akt (Ser473) (Cell Signaling Technology, #4060, 1:1,000), phospho-Src (Tyr416) (Cell Signaling Technology, #2101, 1:1,000), Cxcl9 (R&D System, #AF-492-NA, 1:2,000), Runx2 (Bioworld Technology, #BS8734, 1:1,000), osteocalcin (Santa Cruz Biotechnology, #sc-23790, 1:2,000), phospho-STAT1(Y701) (Abclonal Technology, #AP0135, 1:1,000), phospho-STAT1(S727) (Abclonal Technology, #AP0453, 1:1,000), mTOR ((Cell Signaling Technology, #2983, 1:1,000) and STAT1 (Proteintech Group, #10144-2-AP, 1:1,000). The membrane was then visualized by enhanced chemiluminescence (ECL Kit, Amersham Biosciences). Uncropped western blots scans are provided in the Supplementary Fig. 10a–k.

### Real-time quantitative PCR and microarray analysis

Total RNA was isolated from cell pellets with TRIzol Reagent (Life Technologies, #15596-018) and reverse transcribed (2.5 μg per sample in a 50 μl reaction volume) using PrimeScript Reverse Transcriptase according to the manufacturer's protocol (Takara, #2680B). A volume of 2μl of cDNA (corresponding to 100 ng of total RNA) was used for real-time PCR using SYBR Premix Ex Taq (Takara, #RR420A). Primers for *VEGF*, *Cxcl9* and *STAT1* were as follows: *VEGF* forward, 5′-CCACGTCAGAGAGCAACATCA-3′ and reverse, 5′-TCATTCTCTCTATGTGCTGGCTTT-3′; *Cxcl9* forward, 5′-GGAGTTCGAGGAACCCTAGTG-3′ and reverse 5′-GGGATTTGTAGTGGATCGTGC-3′; *STAT1* forward, 5′-TCACAGTGGTTCGAGCTTCAG-3′ and reverse, 5′-GCAAACGAGACATCATAGGCA-3′.

For mRNA array assay, samples were submitted to Shanghai Biotechnology Corporation for hybridization on an Agilent-014868 Whole Mouse Genome Microarray 4x44K G4122F (Probe Name version). Each microarray chip was hybridized to a single sample labelled with Cy3. Background subtraction and normalization were performed. Finally, mRNAs with expression levels differing by at least threefold between control and Δ*Tsc1* osteoblasts were selected (*P*<0.05). Microarray data have been deposited in GEO database under accession code GSE74781.

### Co-immunoprecipitation assay

Recombinant mouse Cxcl9 (PrimeGene Bio-Tech, #221-09, 50 ng ml^−1^) and VEGF_164_ (R&D System, #493-MV-025, 30 ng ml^−1^) were mixed in ice-cold lysis buffer (40 mM HEPES (pH 7.4), 2 mM EDTA, 10 mM pyrophosphate, 10 mM glycerophosphate, 0.3% CHAPS and one tablet of EDTA-free protease inhibitors (Roche, Basel, Switzerland) per 25 ml). The mixture were then incubated with anti-Cxcl9 antibody (R&D System, #AF-492-NA, 1:500) for 2 h at 4 °C, followed by addition of 30 μl of 50% slurry of protein G Sepharose beads for another 1 h. Beads were then washed four times with lysis buffer, transferred into 2 × SDS sample buffer, boiled for 5 min at 100 °C and subjected to western blot assay for VEGF.

### In vitro kinase assay for mTORC1

Primary calvarial cells were lysed in ice-cold buffer (40 mM HEPES (pH 7.4), 2 mM EDTA,10 mM pyrophosphate, 10 mM glycerophosphate, 0.3% CHAPS and one tablet of EDTA-free protease inhibitors (Roche, Basel, Switzerland) per 25 ml). Supernatants were incubated with anti-mTOR antibody for 2 h at 4 °C, followed by addition of 30 μl of 50% slurry of protein G Sepharose beads for another 1 h. Beads were then washed four times with lysis buffer and once with kinase buffer (25 mM HEPES (pH7.4), 50 mM KCl, 10 mM MgCl_2_ and 250 μM ATP). A unit of 0.4 μg of recombinant glutathione *S*-transferase-tagged full-length STAT1 peptide was added to 30 μl kinase buffer. Kinase assays were performed for 30 min at 30 °C, and terminated by the addition of the 2 × SDS sample buffer followed by boiling for 5 min.

### Binding assay

Iodination of mouse recombinant VEGF_164_ was performed using iodogen (Pierce, Rockford, IL) according to the manufacturer's indications. The specific activities of the ^125^I–VEGF_164_ were about 10^5^ c.p.m. ng^−1^. Confluent HUVECs grown in 24-well dishes were washed twice with ice-cold PBS before binding and incubated with the indicated concentrations of ^125^I–VEGF_164_ and Cxcl9 in DMEM containing 20 mmol l^−1^ HEPES (pH 7.4) and 0.15% gelatin for 2 h at 4 °C. Nonspecific binding of ^125^I–VEGF_164_ was determined in the presence of 1 μg ml^−1^ VEGF_164_. At the end of the binding the cells were washed with and lysed using 0.5 M NaOH. Samples were counted in a γ-counter (Saint-Quentin-Yvelines, France). Specific binding was determined by substracting nonspecific binding from total binding.

### Electrophoretic mobility shift assay

A total of 2 μg of nuclear protein extracted from primary calvarial cells was incubated with a biotin-labelled STAT1-binding-site DNA probe in binding buffer (EMSA kit; Thermo Scientific) for 30 min at room temperature. The probe used for the reaction contained the STAT1-binding site of the *Cxcl9* promoter (γ-RE1 site) with a sequence of 5′-CCTTACTATAAACTCC-3′. After incubation, the samples were separated on a 6% polyacrylamide gel in trisborate EDTA, transferred onto a nylon membrane and fixed on the membrane by ultraviolet crosslinking. The biotin-labelled probe was detected with streptavidin-horseradish peroxidase (EMSA kit; Thermo Scientific). A probe lacking nuclear extracts was used as a negative control. The specificity of the identified STAT3-DNA binding activity was confirmed using a 200-fold excess of unlabelled probe containing the same sequence. For supershift analysis, 1 μg STAT1 antibody (Proteintech) was incubated with nuclear extracts for 30 min before the addition of the biotin-labelled DNA probe.

### siRNA knockdown

We transiently transfected cells with *STAT1* siRNA using Lipofectamine RNAi MAX (Invitrogen, Carlsbad, CA, USA) in Opti-MEM medium (Invitrogen), according to the manufacturer's instructions. The efficiency of transfection was measured by western blot. The sequence of *STAT1* siRNA was as follows: 5′-AAGGAAAAGCAAGCGTAATCT-3′ (GenePharma, Shanghai, China)^43^.

### Statistics

All results are presented as the mean±s.d. Curve analysis was determined using Prism (GraphPad). The data in each group were analysed using the unpaired, two-tailed Student's *t*-test. The level of significance was set at *P*<0.05.

### Data availability

All data generated or analysed during this study are included in this published article and its Supplementary Information files and available from the corresponding author on request.

## Additional information

**How to cite this article:** Huang, B. *et al*. Osteoblasts secrete Cxcl9 to regulate angiogenesis in bone. *Nat. Commun.* **7,** 13885 doi: 10.1038/ncomms13885 (2016).

**Publisher's note**: Springer Nature remains neutral with regard to jurisdictional claims in published maps and institutional affiliations.

## Supplementary Material

Supplementary Information Supplementary Figures
