# Author Correction: Osteoblasts secrete Cxcl9 to regulate angiogenesis in bone

**DOI:** 10.1038/s41467-023-42299-y

**Published:** 2023-10-19

**Authors:** Bin Huang, Wenhao Wang, Qingchu Li, Zhenyu Wang, Bo Yan, Zhongmin Zhang, Liang Wang, Minjun Huang, Chunhong Jia, Jiansen Lu, Sichi Liu, Hongdong Chen, Mangmang Li, Daozhang Cai, Yu Jiang, Dadi Jin, Xiaochun Bai

**Affiliations:** 1 Academy of Orthopedics, Guangdong Province, Department of Orthopedics, The Third Affiliated Hospital, Southern Medical University, Guangzhou, 510630 China; 2 State Key Laboratory of Organ Failure Research, Department of Cell Biology, School of Basic Medical Science, Southern Medical University, Guangzhou, 510515 China; 3 Department of Pharmacology and Chemical Biology, University of Pittsburgh School of Medicine, Pittsburgh, Pennsylvania 15213 USA

Correction to: *Nature Communications* 10.1038/ncomms13885, published online 14 December 2016

The original version of this article contained errors in Figures 5b, 7a and in Supplementary Figure 8a, which occurred during the assembly of the figures.

In figure 5b, the ‘ΔR’ group was a shifted field view of the image in the ‘ΔT+Ab’ group, and the ‘ΔR+Cxcl9’ panel was a shifted field view of the image in from ‘ΔT’ group. The correct version of Figure 5 is:


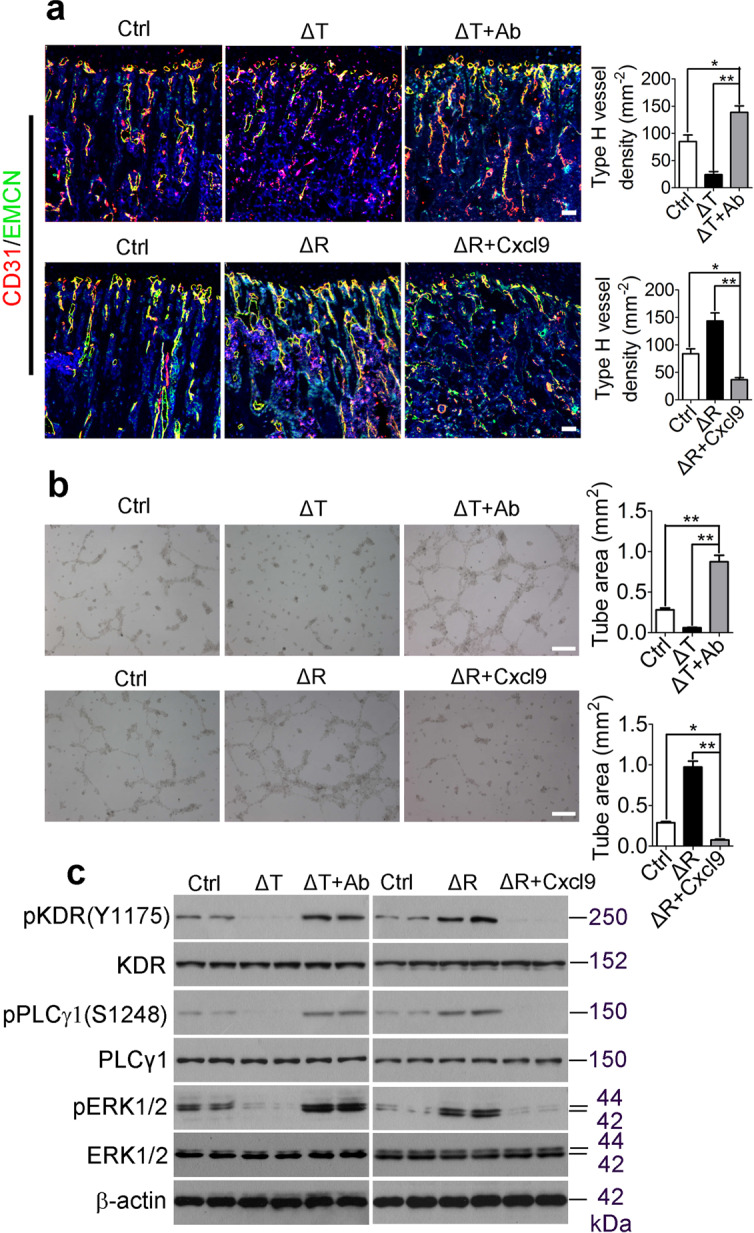


In Figure 7a, the image for the group ‘Cxcl9+VEGF’ was a shifted field view from ‘VEGF’ group in Figure 6a. The correct version of Figure 7 is:


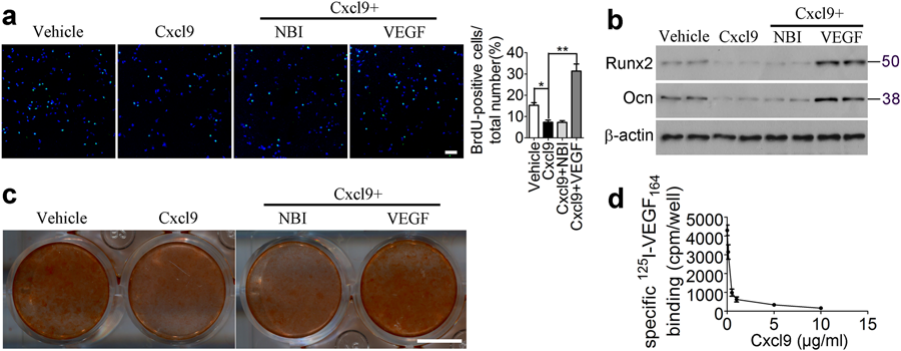


Figures 5 and 7 have been corrected in the original article.

In Supplementary Figure 8a, the image for the group ‘Cxcl9+NBI’ was a shifted field view from ‘Cxcl9’ group. The HTML has been updated to include a corrected version of the Supplementary Information.

### Supplementary information

Updated Supplementary Information

