# Supplementary material for: Osteoblasts secrete Cxcl9 to regulate angiogenesis in bone
Source: Nat Commun. 2016 Dec 14;7:13885. doi: 10.1038/ncomms13885 (PMC5171795; doi:10.1038/ncomms13885)
Supplement: Supplementary Information — Supplementary Figures [file ncomms13885-s1.pdf]

SUPPLEMENTARY INFORMATION

Supplementary Figures

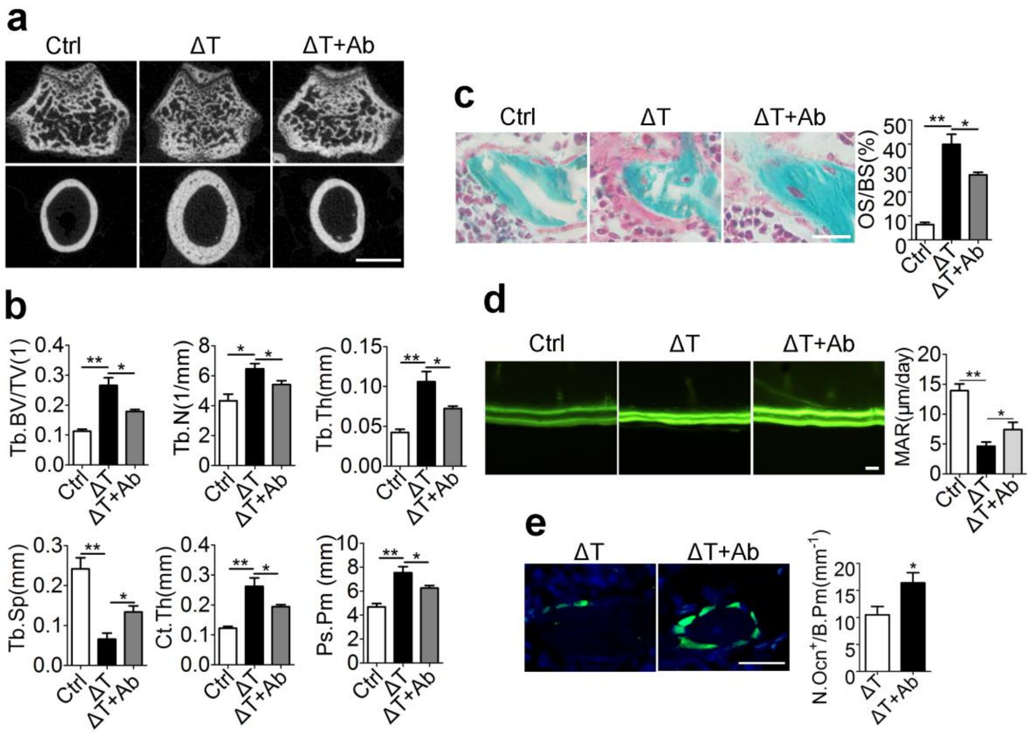

Supplementary Figure 1 Anti-Cxcl9 partially reversed the high volume of immature woven bone in Δ*Tsc1* mice. (a) Representative micro-computed tomography (μCT) images of metaphyseal trabecular bone and cortical bone in the distal femur of 12-week-old male mice bone. Scale bar, 1 mm. (b) Quantitative μCT analysis of the trabecular bone fraction (Tb. BV/TV), trabecular number (Tb. N), trabecular thickness (Tb. Th), trabecular separation (Tb.Sp), cortical thickness (Ct. Th) and periosteal perimeter (Ps. Pm). n=5 per group. （c）Goldner's Masson trichrome staining showed osteoid (red) in mice bone. OS/BS, osteoid per bone surface. Scale bar, 50 μm. (d) Calcin double labeling of mice femoral cortical bone. MAR, mineral apposition rate. Scale bar, 100 μm. (e) Number of osteocalcin positive (N.Ocn^{+}) on the bone surface was measured as cells per millimeter of perimeter in sections (/B.Pm). Scale bar, 50 μm. Data are shown as mean ± s.d. *P < 0.05, **P < 0.01 (Student's t-test).

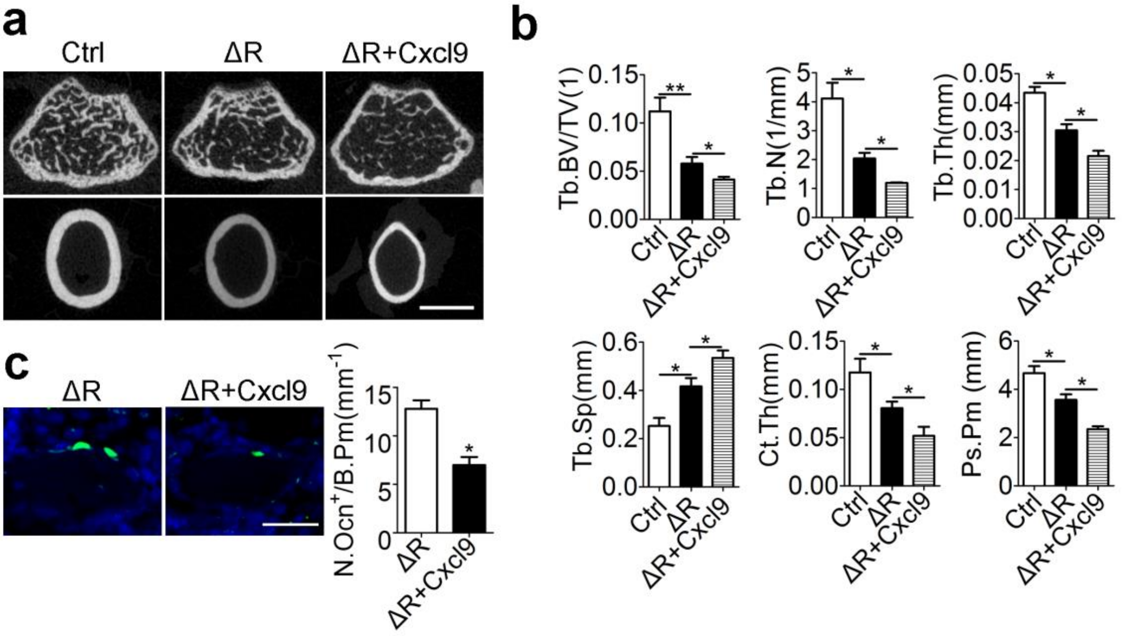

Supplementary Figure 2 Cxcl9 caused further loss of bone mass in ΔRaptor mice (a) Representative micro-computed tomography (μCT) images of metaphyseal trabecular bone and cortical bone in the distal femur. Scale bar, 1 mm. (b) Quantitative μCT analysis of the trabecular bone fraction (Tb. BV/TV), trabecular number (Tb. N), trabecular thickness (Tb. Th), trabecular separation (Tb.Sp), cortical thickness (Ct. Th) and periosteal perimeter (Ps. Pm). n=5 per group. (c) Number of osteocalcin positive ($N.Ocn^+$) on the bone surface was measured as cells per millimeter of perimeter in sections (/B.Pm). Scale bar, 50 μm. Data are shown as mean ± s.d. *$P < 0.05$, **$P < 0.01$ (Student's t-test).

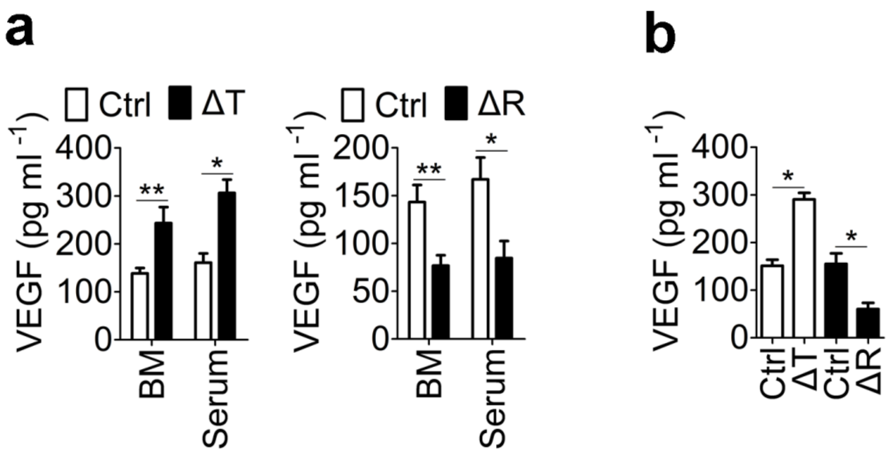

Supplementary Figure 3 Secretion of VEGF is elevated in Δ*Tsc1* osteoblasts and decreased in Δ*Raptor* osteoblasts. (a) ELISA analysis of VEGF concentrations in bone marrow (BM) and serum of mice. (b) VEGF concentrations in conditional medium from cultured primary osteoblasts. n=5 per group. Data are shown as mean ± s.d. $*P < 0.05$, $**P < 0.01$ (Student's t-test).

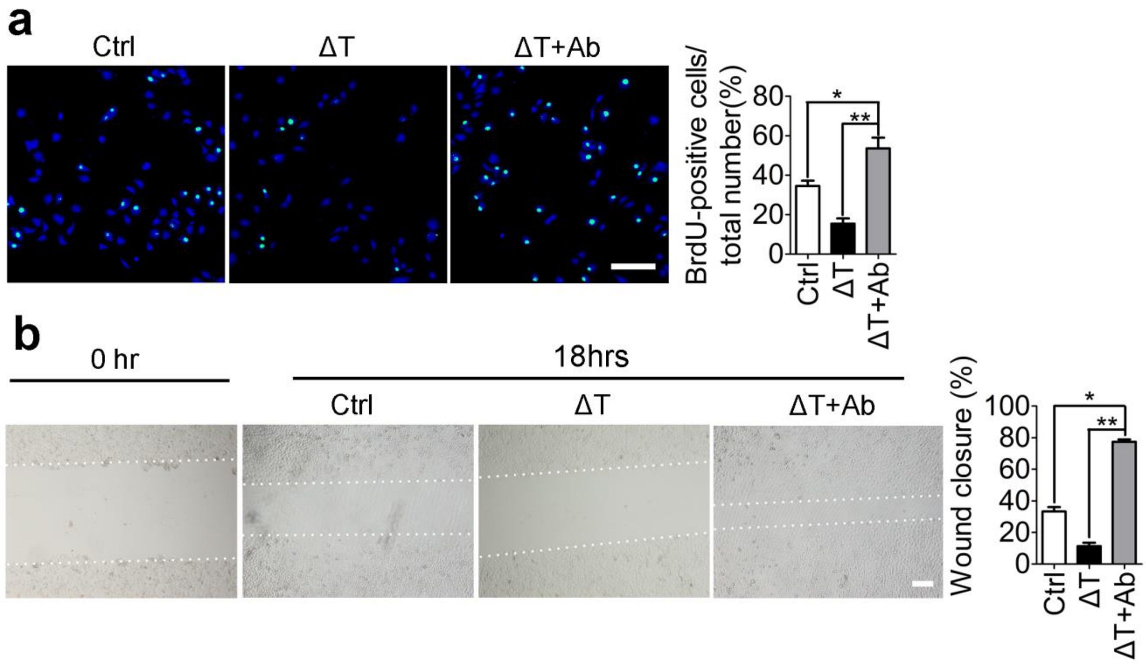

Supplementary Figure 4 Cxcl9 antibody promotes proliferation and migration of HUVECs maintained in CM from Δ*Tsc1* osteoblasts. (a) Representative confocal images of immunostaining of BrdU (green) in HUVECs and quantitative analysis of $BrdU^+$ cells compared with total cells. Scale bar, 50 μm. n=9 per group. (b) Representative photomicrographs of wounds in HUVECs at 0 h and after 18 h; dotted lines highlight the linear scratch/wound for each group of cells. The bar graph shows the mean percentage wound closure. Scale bar, 200 μm. n=9 per group. Data are shown as mean ± s.d. *P < 0.05, **P < 0.01 (Student's t-test).

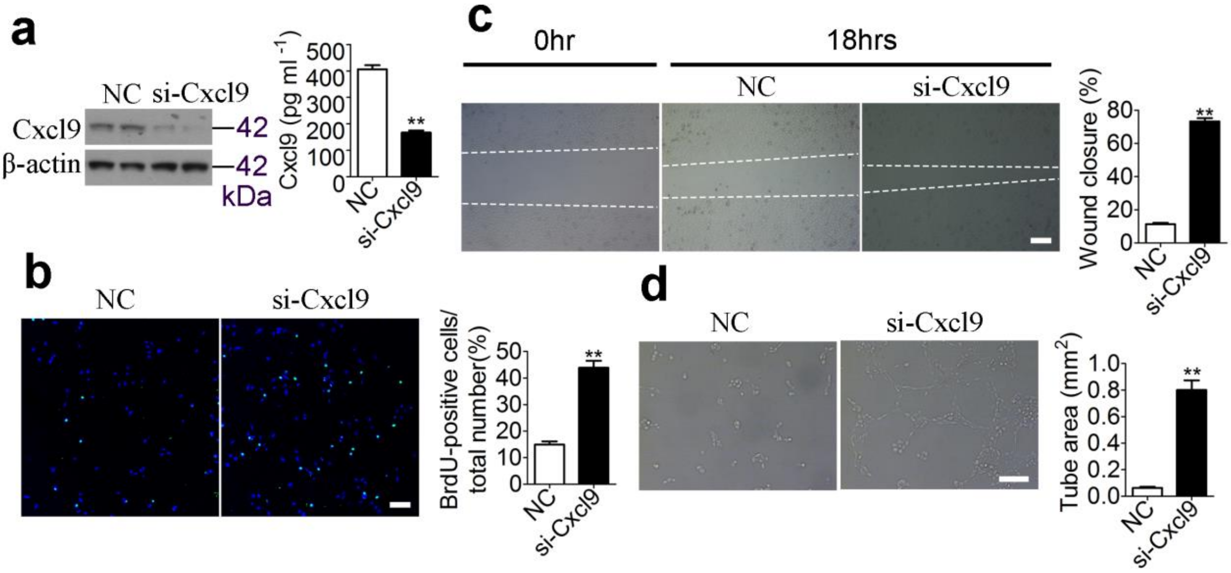

Supplementary Figure 5 Cxcl9 expression was interfered in Δ*Tsc1* osteoblasts by siRNA, CM from which promoted proliferation, migration and tube formation of HUVECs. (a) Western blot and ELISA assay showed efficient Cxcl9 deletion in Δ*Tsc1* osteoblasts by siRNA. (b) Representative confocal images of immunostaining of Brdu (green) in HUVECs and quantitative analysis of $Brdu^+$ cells compared with total cells. Scale bar, 100 μm. n=9 per group. (c) Representative photomicrographs of wounds in HUVECs at 0 h and after 18 h; dotted lines highlight the linear scratch/wound for each group of cells. The bar graph shows the mean percentage wound closure. Scale bar, 200 μm. n=9 per group. (d) Representative Matrigel tube formation assay images and quantitative analysis of tube area in cultures of HUVECs. Scale bar, 100 μm. n=9 per group. Data are shown as mean ± s.d. **P < 0.01 (Student’s t-test).

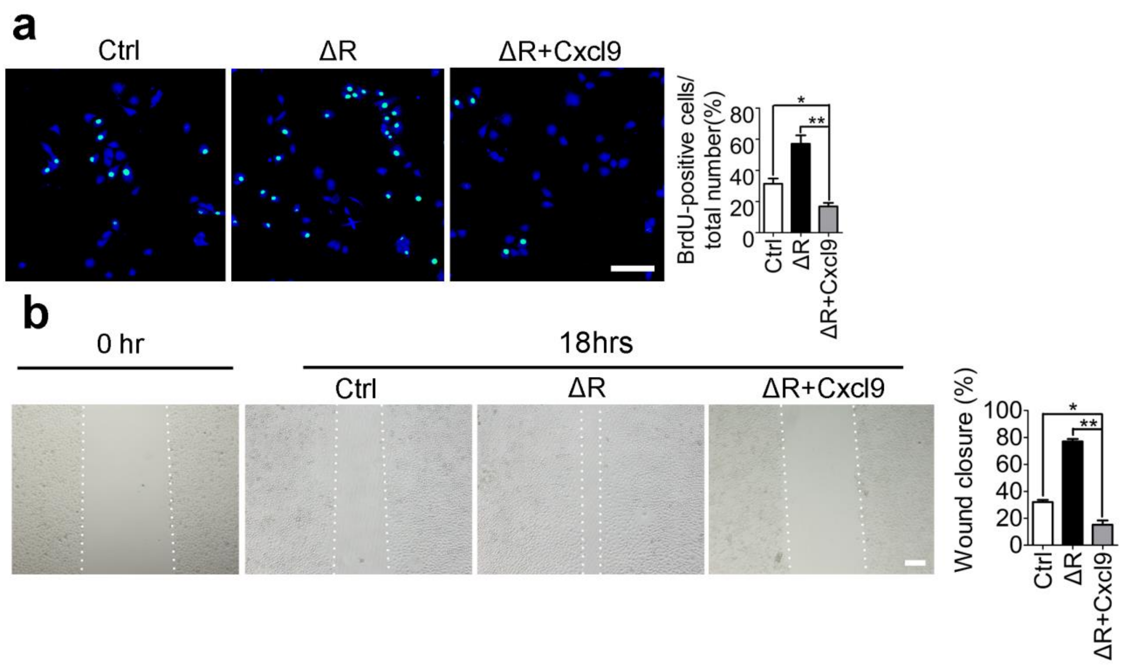

Supplementary Figure 6 Cxcl9 inhibits proliferation and migration of HUVECs maintained in CM from ΔRaptor osteoblasts. (a) Representative confocal images of immunostaining of BrdU (green) in HUVECs and quantitative analysis of $BrdU^+$ cells compared with total cells. Scale bar, 50 μm. n=9 per group. (b) Representative photomicrographs of wounds in HUVECs at 0 h and after 18 h; dotted lines highlight the linear scratch/wound for each group of cells. The bar graph shows the mean percentage wound closure. Scale bar, 200 μm. n=9 per group. Data are shown as mean ± s.d. *P < 0.05, **P < 0.01 (Student's t-test).

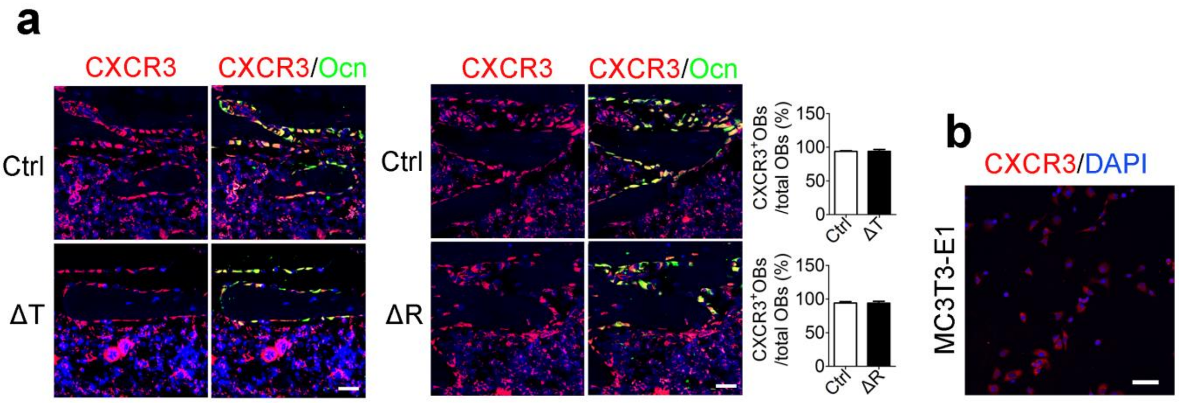

Supplementary Figure 7 CXCR3 expression in osteoblasts. (a) Osteoblasts showed consistent CXCR3 expression in the two knockout mouse models. Scale bar, 50 μm. n=9 per group. Data are shown as mean ± s.d.(b) The majority of MC3T3-E1 cells exhibit CXCR3 expression.

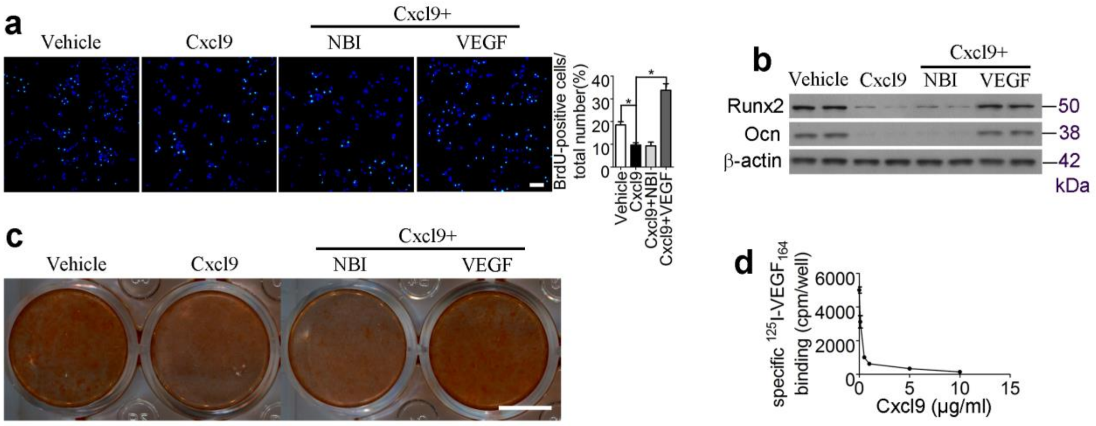

Supplementary Figure 8 Cxcl9 suppresses osteogenesis of BMSCs by interacting with VEGF and abrogating binding of VEGF to the cells. (a) BrdU staining of BMSCs and quantitative analysis of $BrdU^+$ cells out of total cells. Scale bar, 100 μm. (b) Western blot analysis of osteoblastic marker Ocn and Runx2 expression in BMSCs cells on the 7th day of osteogenic induction. (c) Alizarin red staining of differentiated BMSCs on the 14th day. Scale bar, 1 cm. (d) Binding of ^{125}I–$VEGF_{164}$ to BMSCs in the presence of increasing concentrations of Cxcl9. Shown is the specific binding which was calculated by subtracting the nonspecific binding from the total binding. Data are shown as mean ± s.d. *$P < 0.05$ (Student's t-test).

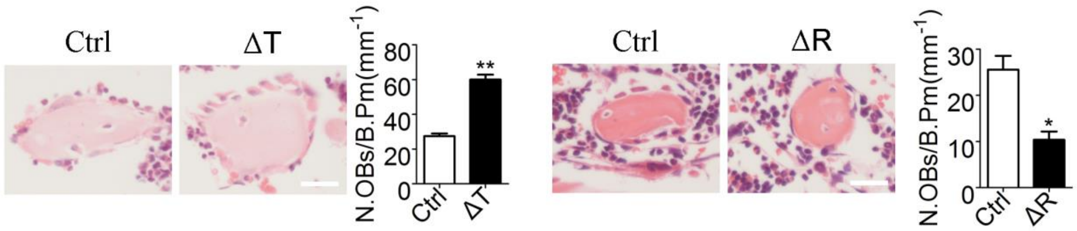

Supplementary Figure 9 Numbers of osteoblasts per bone perimeter in the two transgenic mouse models. Bone slices were stained with H&E and osteoblasts on bone surface were discerned by morphology and measured as cells per millimeter of perimeter in sections (/B.Pm) by two independent observers blinded to the groups. Scale bar, 50 μm. Data are shown as mean ± s.d. *P < 0.05, **P < 0.01 (Student's t-test).

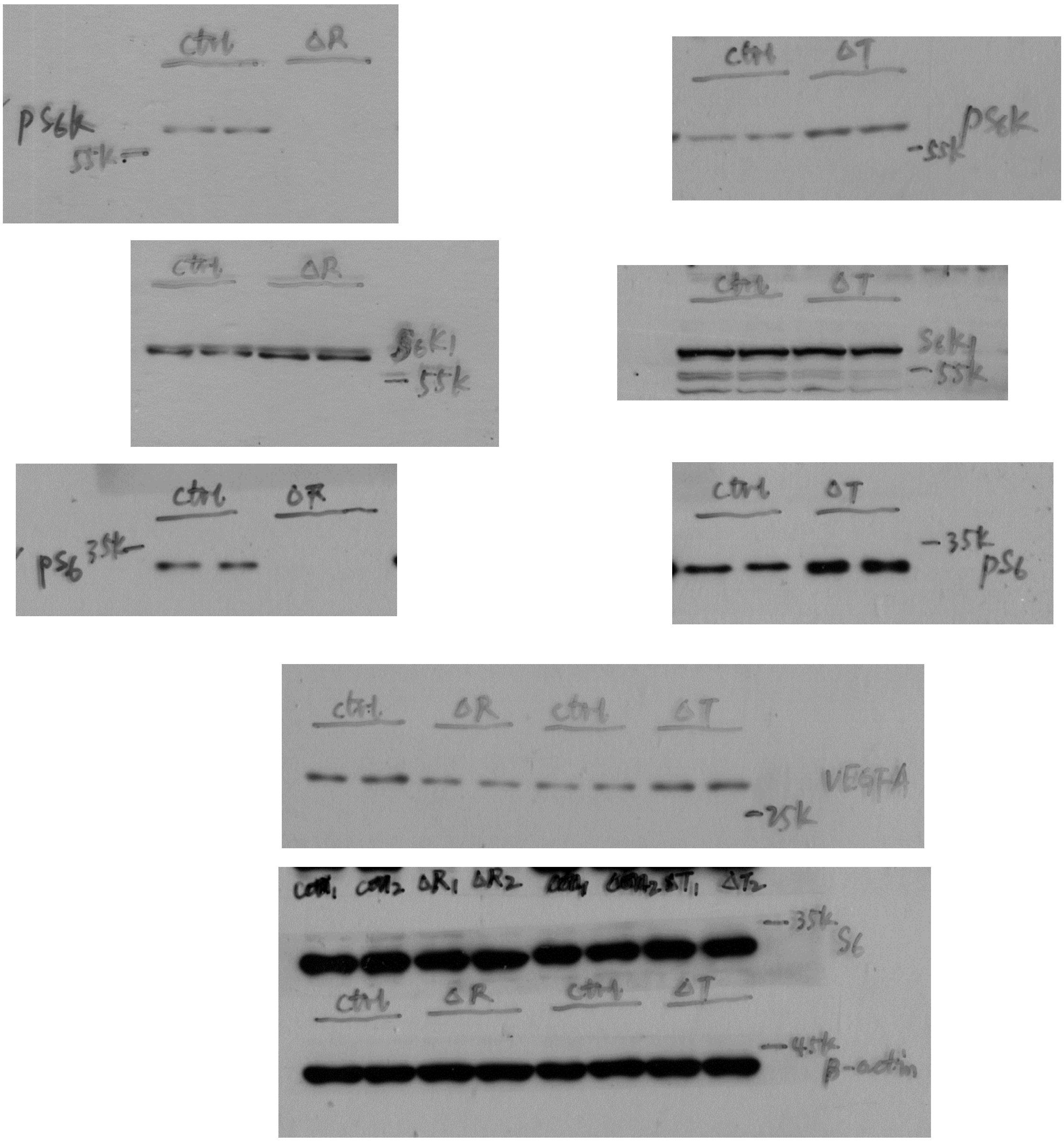

Supplementary Figure 10a. Uncropped pictures of Western blots results in Figure 3c.

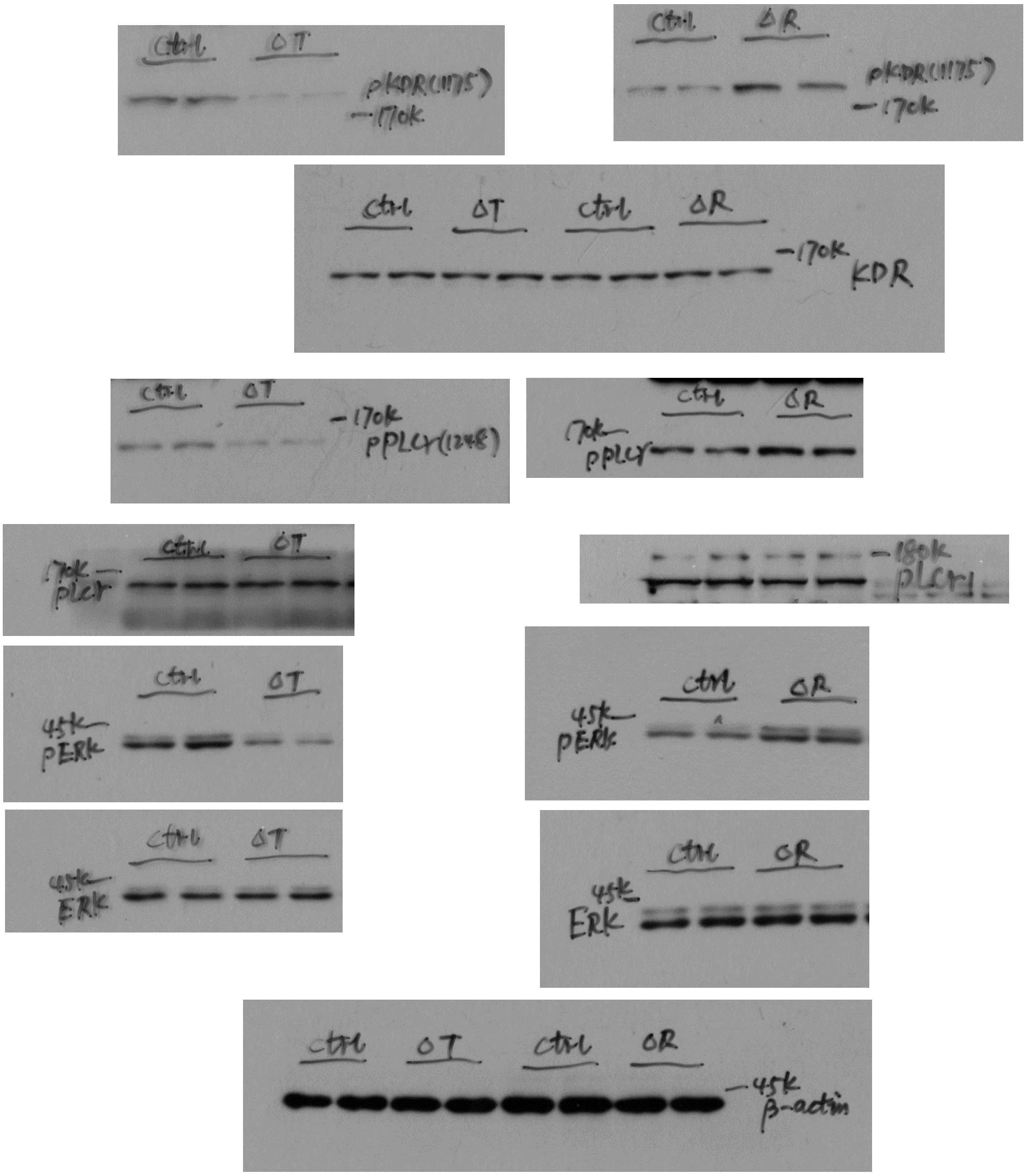

Supplementary Figure 10b. Uncropped pictures of Western blots results in Figure 3d.

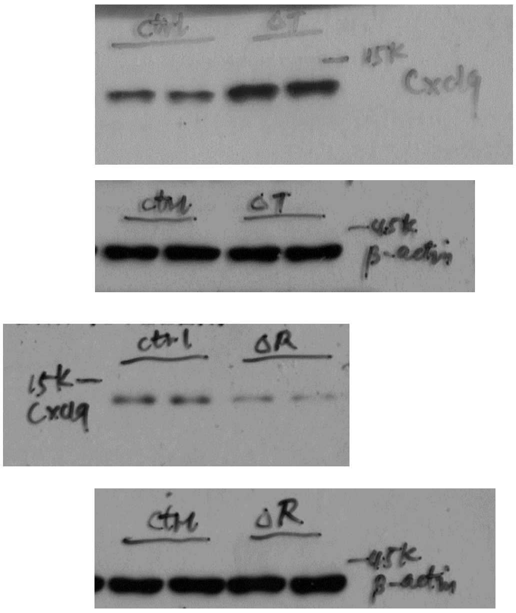

Supplementary Figure 10c. Uncropped pictures of Western blots results in Figure 4f.

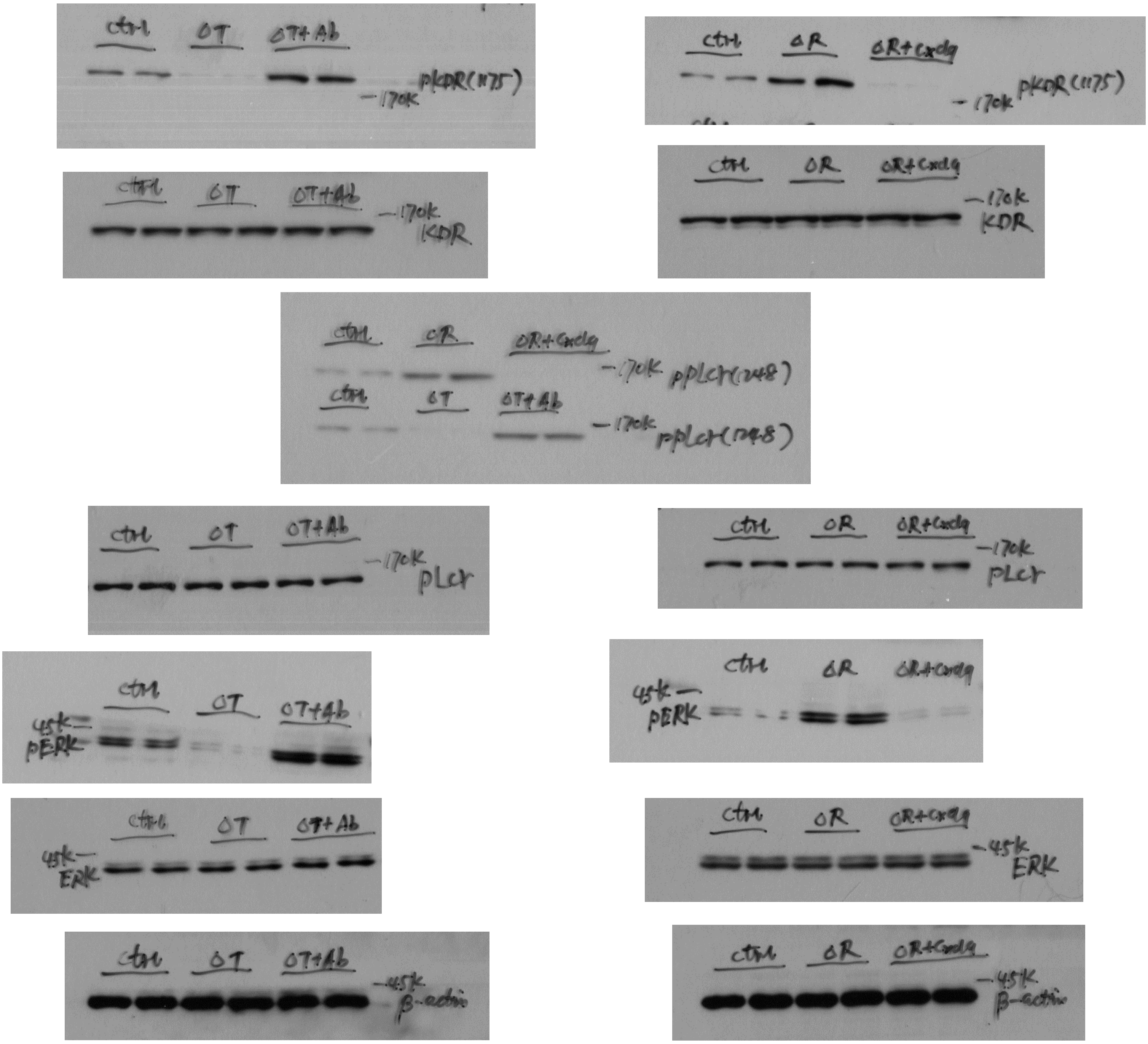

Supplementary Figure 10d. Uncropped pictures of Western blots results in Figure 5c.

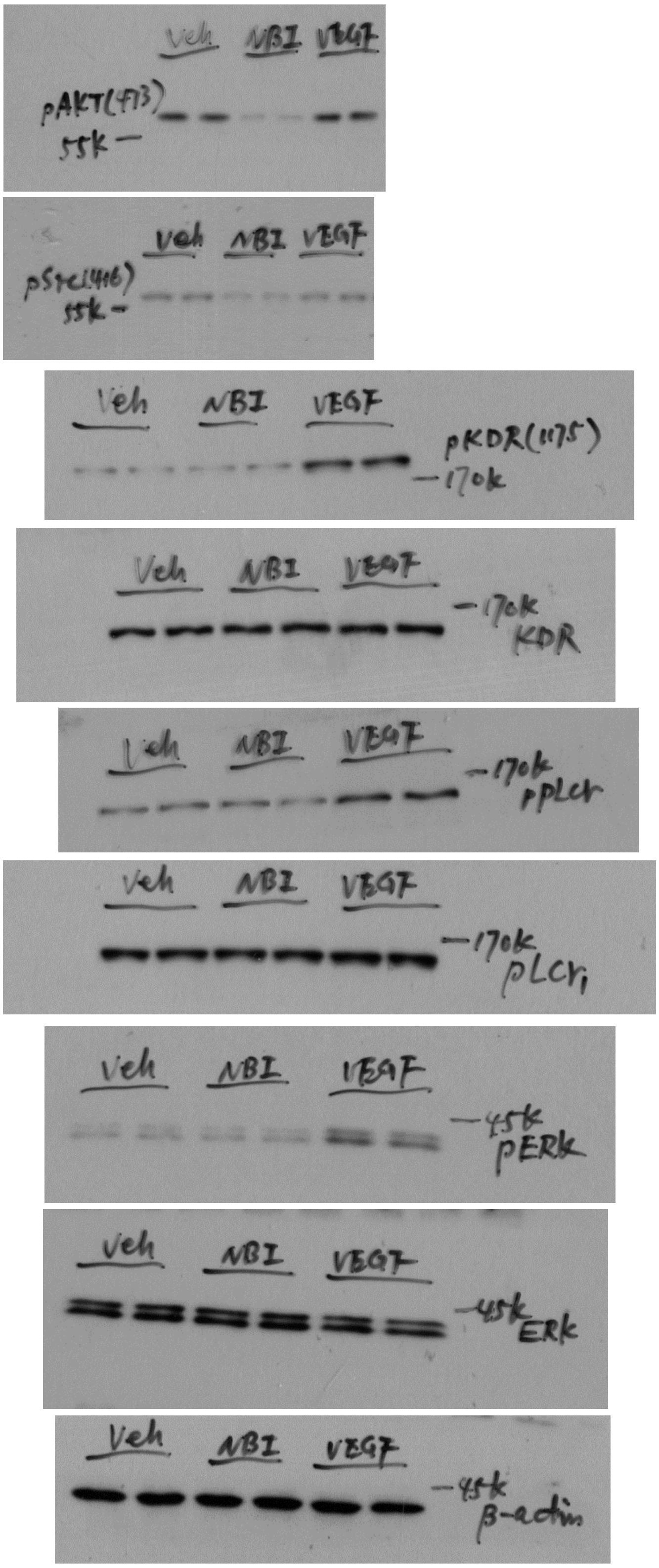

Supplementary Figure 10e. Uncropped pictures of Western blots results in Figure 6d.

For Figure 6g

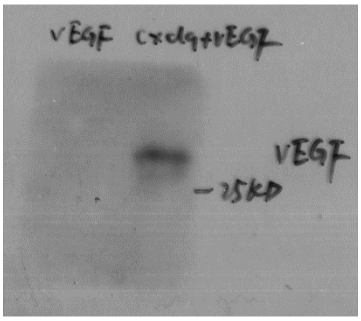

For Figure 7b

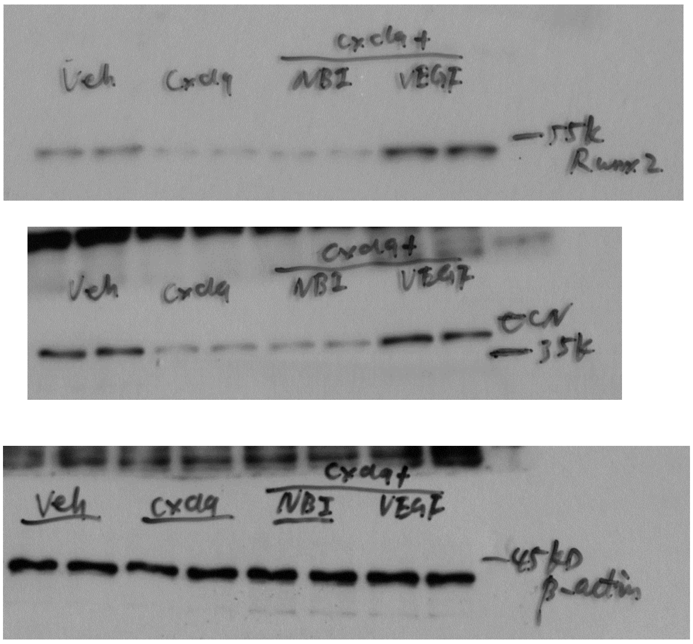

Supplementary Figure 10f. Uncropped picture of Western blots results in Figure 6g and Figure 7b.

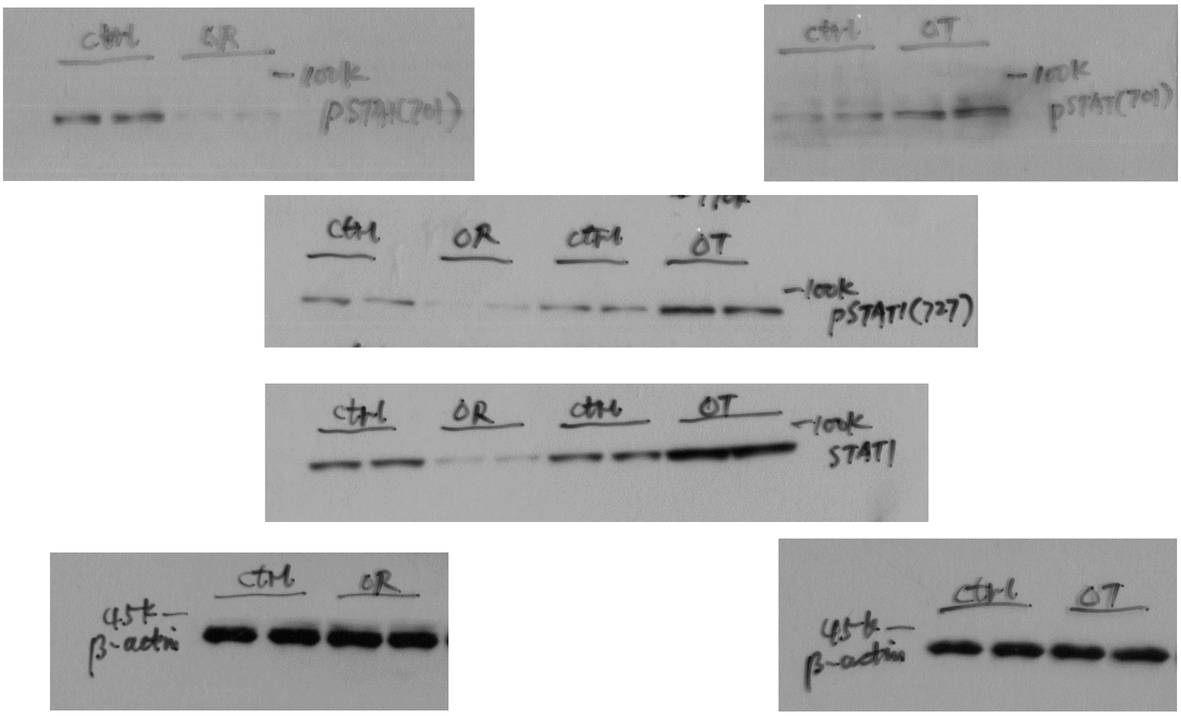

Supplementary Figure 10g. Uncropped picture of Western blots results in Figure 8b.

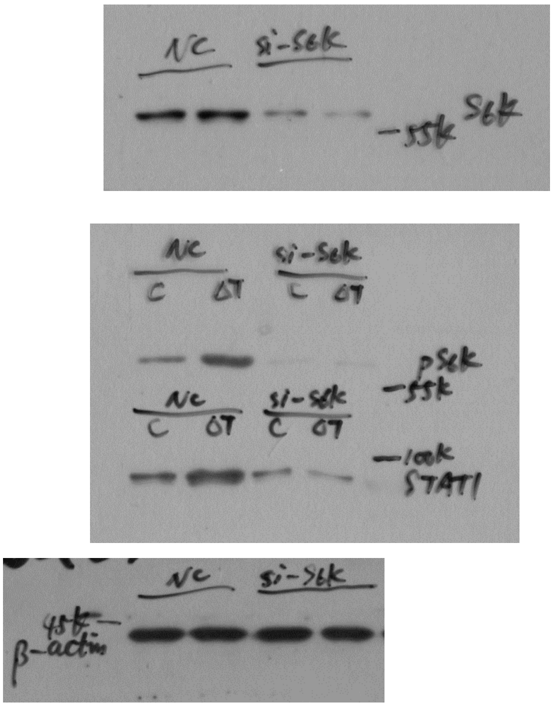

Supplementary Figure 10h. Uncropped pictures of Western blots results in Figure 8d.

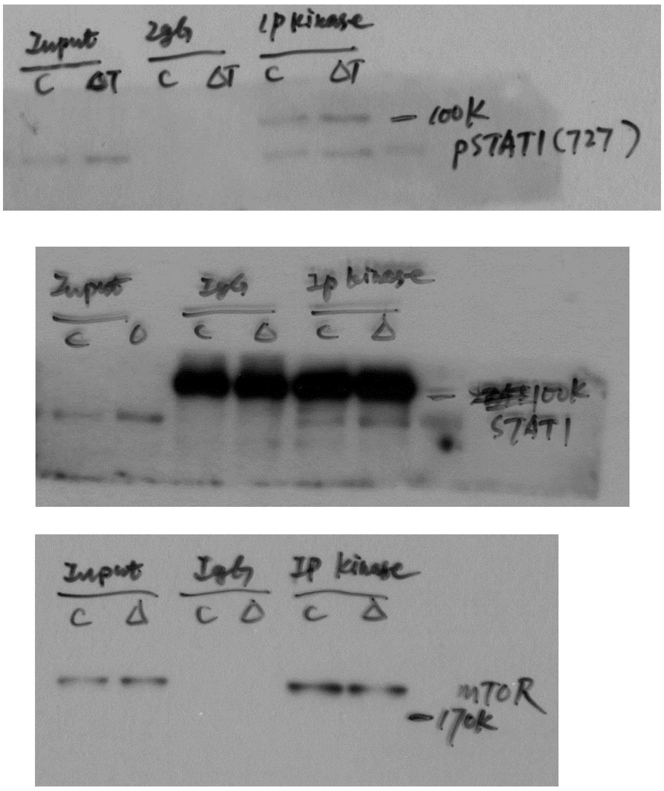

Supplementary Figure 10i. Uncropped pictures of Kinase assay results in Figure 8e.

For Figure 8f

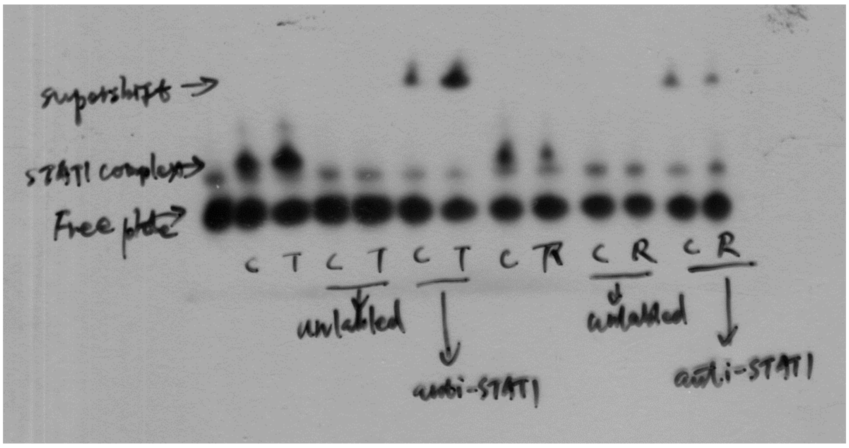

For Figure 8g

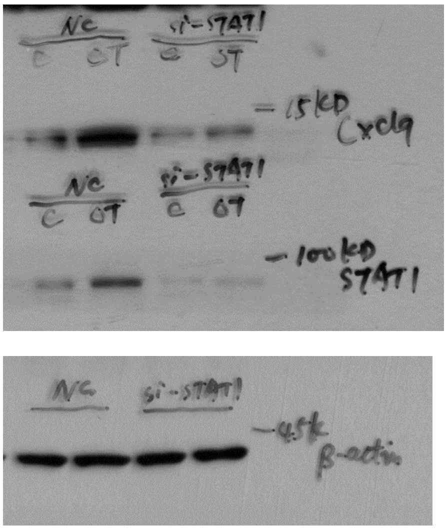

Supplementary Figure 10j. Uncropped picture of EMSA and Western blots results in Figure 8f, g.

For Supplementary Figure 5a

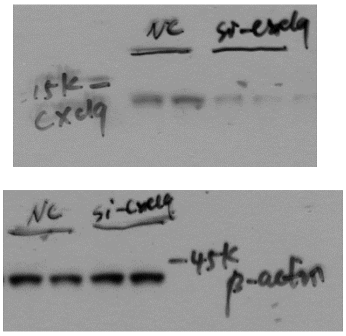

For Supplementary Figure 8b

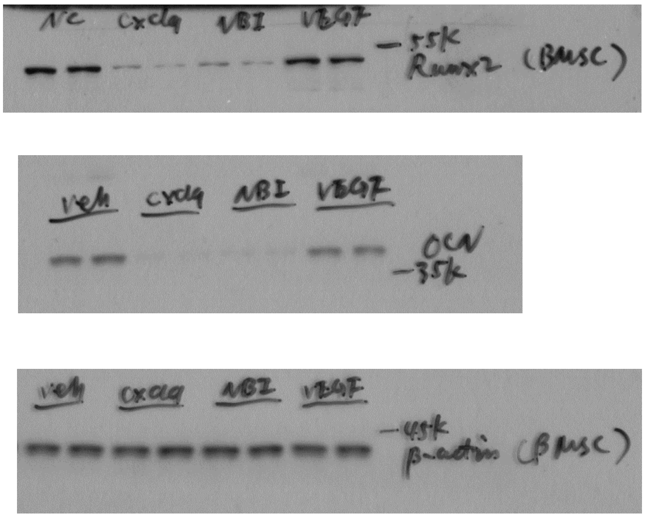

Supplementary Figure 10k. Uncropped picture of Western blots results in Supplementary Figure 5a, 8b.
